# Identification of sex-linked molecular markers in the Asiatic toad (*Bufo gargarizans* Cantor, 1842) reveals an XX/XY sex determination system

**DOI:** 10.1186/s12864-026-13266-w

**Published:** 2026-08-14

**Authors:** Jun Ping, Lulu Shi, Ziruo Huang, Lei Xie, Yongpu Zhang

**Affiliations:** 1 Quzhou People’s Hospital, Quzhou Affiliated Hospital of Wenzhou Medical University, Quzhou, Zhejiang 324000 China; 2 Quzhou Foreign Language School, Quzhou, Zhejiang 324000 China; 3 College of Life and Environmental Science, Wenzhou University, Wenzhou, Zhejiang 325035 China; 4 Zhejiang Provincial Key Laboratory for Subtropical Water Environment and Marine Biological Resources Protection, Wenzhou University, Wenzhou, Zhejiang 325035 China

**Keywords:** Sex-linked markers, Sex determination system, Sex chromosome, Sex chromosome turnover, *DMRT1*

## Abstract

**Background:**

Previous karyotypic analyses of *Bufo gargarizans* reported 11 pairs of homomorphic chromosomes (2n = 22) and no cytologically distinguishable sex chromosomes. A ZW-type sex determination system had been hypothesized based on differential replication patterns in chromosome pair 10, but this remained speculative in the absence of molecular evidence.

**Result:**

Here, we identified male-specific genetic markers, providing the first definitive molecular evidence for an XY-type sex determination system in this species. Chromosomal localization of these markers confirmed chromosome 1 as the sex chromosome. By calculating *Fst* between males and females, we delineated the non-recombining region (NRR) on chromosome 1, which spans approximately 212 Mb (246.69 Mb to 458.35 Mb), revealing a remarkably large sex-differentiating region with low recombination rates. We further detected non-homologous sex chromosome transitions between *B. gargarizans* and *B. bufo*, highlighting the dynamic evolution of sex chromosomes within the genus *Bufo*.

**Conclusion:**

These findings revise the current understanding of sex determination in *B. gargarizans*, define the extent of the NRR, and establish this species as a valuable model for studying the origin and differentiation of sex chromosomes, thereby contributing important insights into the diversity of sex-determining mechanisms in amphibians and vertebrates.

**Supplementary Information:**

The online version contains supplementary material available at https://doi.org/10.1186/s12864-026-13266-w.

## Introduction

Sex determination in vertebrates represents a remarkably diverse biological process, fundamental to reproductive success and genetic diversity [1–3]. This system exhibits profound evolutionary plasticity, spanning a spectrum from strictly genetic sex determination (GSD) to environmental sex determination (ESD), as well as complex intermediate modes [3–6]. Elucidating these mechanisms is critical not only for reconstructing the evolutionary trajectory of vertebrate reproduction but also for understanding how species adapt to shifting environmental pressures [1, 7].

The evolution of sex chromosomes is a fundamental driver of plasticity in sex determination systems [8–10]. The classical paradigm posits that sex chromosomes emerge from autosomes through the acquisition of a sex-determining locus, followed by the progressive expansion of recombination suppression. This suppression facilitates the linkage of sexually antagonistic or beneficial alleles, ultimately precipitating the degeneration of the non-recombining Y or W chromosome [9–13]. However, emerging evidence challenges this unidirectional model, suggesting a more intricate evolutionary trajectory. Frequent turnovers of sex-determining regions can fundamentally reshape genetic architecture, modulate recombination landscapes, and renew the genomic context for sexual selection [14–17]. A compelling example is found in *Amolops* frogs, where research has unveiled heterogeneous sex chromosome evolution involving two distinct chromosome pairs. In these species, the observed homomorphy of sex chromosomes stems from a combination of sex chromosome turnover and ongoing recombination between X and Y chromosomes. Notably, this persistent X-Y recombination within homologous regions serves as a critical mechanism that prevents sex chromosome differentiation [18].

In amphibians, nearly 96% of species possess homomorphic sex chromosomes [19–21], which typically exhibit limited degeneration of the Y (or W) chromosome [22–24]. This morphological characteristic has long posed significant challenges for the study of sex determination systems in this group [25]. Even among the few amphibian species with morphologically distinguishable heteromorphic sex chromosomes [21, 26], identifying the heterogametic sex using classical cytogenetic methods remains a time-consuming and resource-intensive task [27–30]. Historically, some researchers even posited that amphibians lacked sex chromosomes [31]. However, the continuous advancement of next-generation sequencing (NGS) technologies has provided new breakthroughs for screening sex-linked markers [14, 32–35]. The identification of sex-specific loci is crucial for uncovering sex determination systems, understanding their underlying mechanisms, and ultimately pinpointing sex determination genes [10, 25]. For instance, the application of DArTseq technology to *Rana clamitans* has facilitated the development of sex-linked markers, offering insights into the evolution of its sex chromosomes [36]. Similarly, ddRAD-seq was employed to identify sex-specific markers in Boa and Python, revealing for the first time that these species possess XY sex chromosomes [37]. Furthermore, target region amplification polymorphism of *DMRT1* was utilized to screen for sex-linked molecular markers in *Rana dybowskii* populations, confirming male heterogamety [25]. Finally, a simple transcriptome-based protocol was developed for detecting sex-linked markers, circumventing the limitations of traditional methods, as successfully demonstrated in *Carinascincus ocellatus* [38]. These techniques are essential for characterizing sex chromosome systems in non-model species, thereby filling critical gaps in our understanding of sex chromosome evolution across the tree of life.

*Bufo gargarizans* is a widely distributed amphibian species across East Asia, inhabiting most provinces in China (excluding Xinjiang and Ningxia) as well as regions in Russia, Japan, and the Korean Peninsula [39–42]. Historically, this taxon was partitioned into four distinct species: *B. gargarizans*, *B. andrewsi*, *B. minshanicus*, and *B. bankorensis* [40]. However, recent phylogenetic and genomic evidence suggests that these entities do not constitute independent evolutionary lineages [40, 41, 43], leading to a growing consensus that they should be classified as a single species, *B. gargarizans* [39, 40, 44]. Given its expansive geographic range and large effective population size, *B. gargarizans* serves as an ideal model system for investigating the evolutionary history and ongoing dynamics of sex chromosomes in shaping contemporary genetic architecture.

Previous karyotypic analyses of *B. gargarizans* populations have consistently demonstrated a diploid chromosome number of 2n = 22, comprising six pairs of large and five pairs of small chromosomes. Conventional staining and C-banding techniques failed to identify heteromorphic sex chromosomes or distinct sex-differentiation regions [45, 46]. However, subsequent investigations using BrdU-Hoechst-Giemsa staining revealed a differential replication pattern on the 10th chromosome pair: while replication is synchronous in males, it is asynchronous in females. This observation suggested a ZW-type sex determination system, with the 10th chromosome pair potentially serving as the sex chromosomes [47]. Despite these cytogenetic inferences, definitive molecular evidence remains elusive. In this study, we employed double-digest restriction site-associated DNA sequencing (ddRAD-seq) to identify sex-linked molecular markers, aiming to validate the sex determination system, localize the sex chromosomes, and characterize the sex-linked genomic regions in *B. gargarizans*.

## Materials and methods

### Animals and samples

During the 2023 and 2024 breeding season, 42 adult *B. gargarizans* individuals were collected from the vicinity of a reservoir in Quzhou, Zhejiang Province, China (30°57′41.4286″ N, 112°11′03.1067″ E). Among these, 12 individuals (6 females and 6 males) were selected for ddRAD sequencing. An additional 30 individuals (15 females and 15 males) were subsequently used to validate the reliability of the identified sex-specific markers. Sex determination was performed based on secondary sexual characteristics: males were identified by the presence of black nuptial pads on the inner three fingers, while females were confirmed by the visibility of eggs through the abdominal skin [42]. For both ddRAD sequencing and marker validation, a small tissue sample (toe-clip tissue) was collected from each individual in the field and preserved in 95% ethanol. To minimize potential harm, the wound site was disinfected with 70% ethanol prior to the release of the individuals. All animal handling and experimental procedures were approved by the Animal Protection and Utilization Committee of the College of Life and Environmental Sciences, Wenzhou University (Permit No.: wzu-039).

### Library preparation for ddRAD-seq

Genomic DNA was extracted from toe clips using the DNeasy Blood and Tissue Kit (Qiagen, Hilden, Germany) following the manufacturer’s protocol. DNA integrity and concentration were assessed [via 1% agarose gel electrophoresis and Qubit 3.0 fluorometric quantification]. High-quality DNA samples were subjected to double-digest restriction-site associated DNA sequencing (ddRADseq). Briefly, 500 ng of genomic DNA was digested with *EcoRI* and *NlaIII* (New England Biolabs, Ipswich, MA, USA). The resulting fragments were ligated to *EcoRI* and *NlaIII* adapters, the former containing sample-specific barcodes, using T4 DNA ligase (NEB) and ATP. The reaction mixture was incubated at 37 °C for 3 h, followed by enzyme inactivation at 65 °C for 30 min. Ligation products were size-selected (400–600 bp) via agarose gel electrophoresis and purified. The recovered fragments were quantified using a Qubit 3.0 fluorometer (Life Technologies, Carlsbad, CA, USA), pooled in equimolar ratios, and sequenced on an Illumina NovaSeq 6000 platform (150 bp paired-end reads). Detailed experimental procedures are provided in Figure S1.

### *De Novo* assembly and SNP calling

Raw Illumina sequencing data were demultiplexed, trimmed, and filtered using the *process_radtags* module in STACKS (v2.41). This step involved the removal of low-quality reads and those containing ambiguous ‘N’ bases, alongside the validation and correction of restriction enzyme cut sites [48–50].

Subsequently, *de novo* assembly and SNP calling were performed using the *denovo_map.pl* pipeline. The pipeline executed the following sequential modules: *ustacks* for individual-level assembly and SNP identification; *cstacks* for catalog construction; *sstacks* for matching samples against the catalog; *tsv2bam* for reorienting data from sample-centric to locus-centric formats; and *gstacks* for paired-end contig assembly and population-level SNP re-calling [48, 49].

Key parameters for the pipeline were optimized to maximize the recovery of genomic loci: minimum stack depth (m = 2), maximum mismatches between stacks within individuals (M = 2), and maximum mismatches between stacks across individuals (*n* = 1). While lower parameter values were selected to prioritize locus retention, potential artifacts introduced by these settings were mitigated through subsequent stringent filtering criteria [18, 51]. Finally, population-level statistics were generated using the *populations* program [49, 50].

### Sex-linked markers isolation and validating

To identify sex-linked markers, we employed three complementary approaches: the frequency method (i), the heterozygosity method (ii), and the sex-specific method (iii). Sex-linked markers, including SNPs and indels, were derived from the *populations* module in STACKS (v2.41). Specifically, sex-linked SNP loci were identified using the *population.sumstats.tsv* file based on methods (i) and (ii). Indel loci were identified via the *matches.tsv* file (method iii) by screening for loci present exclusively in one sex. These were designated as putative sex-specific markers [14, 18, 33, 34]. For methods (i) and (ii), we considered a locus that was homozygous in all female samples and heterozygous in all male samples as a putative SNP marker for the XX/XY sex determination system (the opposite pattern would indicate a ZW system). In method (iii), we used sex-specific loci: specifically, loci that were completely absent in all females while present in all males were regarded as putative Y-specific loci (again, the opposite pattern would indicate a ZW system). All analyses were performed using custom R scripts, as described in Ping et al. (2022). Finally, to ensure high confidence, we filtered these candidates to generate ‘confirmed’ sex-specific RAD markers. This step excluded any putative markers detected in raw reads from the opposite sex or mapped to the female *B. gargarizans* reference genome (GCF_014858855.1) [14, 18, 33, 34].

To validate the candidate sex-linked markers, we performed PCR-based verification using an independent cohort. Two validation methods were employed. In Test 1, the presence or absence of PCR amplicons was assessed via agarose gel electrophoresis. Authentic sex-linked markers were defined as those showing bands in males but not in females in the gel images. In Test 2, Sanger sequencing was performed to further confirm marker authenticity. Authentic sex-linked markers were defined as those where the sequencing chromatograms revealed consistent heterozygosity (double peaks) at the SNP site in all males, whereas all females exhibited homozygosity (single peaks). Primers were designed using Primer3 [52, 53] to amplify genomic DNA from individuals not included in the original ddRAD-seq analysis, thereby ensuring the reliability and reproducibility of the identified markers.

### Sex chromosome identification

To determine the chromosomal locations of the sex-linked RAD markers identified via ddRAD-seq, we mapped the sequences against the *B. gargarizans* reference genome (GCF_014858855.1) [54] using BLASTn (v2.6.0+). We established a local BLAST database and aligned the sex-linked RAD-tags (SNPs) with an E-value threshold of ≤ 1 × 10⁻²⁰. To ensure alignment specificity, we retained only those hits where the best match had an E-value at least five orders of magnitude lower than the next best hit, this threshold was set to account for the repetitive nature of anuran genomes [14]. Loci that failed to align or exhibited multiple ambiguous best matches were documented for further analysis.

### Validation of sex-linked markers and sex chromosomes

When screening for sex-linked loci in large-scale SNP datasets, mitigating false positives arising from artifacts or stochastic variation is critical. To validate the statistical significance of identified markers, we employed permutation testing. By randomly shuffling sex labels and re-analyzing the data, we constructed a null distribution to assess the probability of observed associations, thereby minimizing spurious correlations potentially driven by limited sample sizes or population structure. Given the absence of a reference genome, we randomly partitioned the 12 samples into two groups of six, irrespective of biological sex, and treated these as artificial sex groups. Following missing data filtering, Fisher’s exact test was applied to identify loci exhibiting significant differentiation between these groups. This procedure was iterated 100 times to establish a confidence interval for the null distribution, which was then integrated with the data in Table 1 for visualization. Notably, this permutation analysis was based on the total count of sex-specific RAD markers rather than ‘validated’ markers, as the latter would necessitate computationally prohibitive re-processing of raw sequencing data. This approach is consistent with established protocols for assessing the significance of RAD-seq results in similar contexts [37].

**Table 1:** Summary of identification results of sex-linked markers in *Bufo gargarizans* from ddRAD-seq datasets

Species	No. of Males	No. of Females	Sex-linked RAD-tags (SNPs) identified (XY / ZW)											No. of PCR Validated Markers (i)(ii)&(iii) (XY)	Sex-Determining System
			Frequency Method (i)		Heterozygosity method (ii)		Sex-specific method (iii)			Confirmed male-specific markers (i)(ii)(XY)	Confirmed male-specific markers (iii)(XY)	Confirmed female-specific markers (i)(ii)(ZW)	Confirmed female-specific markers (iii)(ZW)		
XY			ZW	XY	ZW	XY	ZW								
*Bufo gargarizans*	6	6	443(549)	8(13)	443(549)	15(20)	919		141	245(290)	11	0	0	7&1	XY

To validate the sex chromosomes using the *B. gargarizans* reference genome, we first mapped putative sex-linked markers to the assembly to determine their chromosomal distribution. To establish a null distribution of mapping rates, we performed a permutation test: we randomly sampled subsets of reads from the Stacks locus catalog, matching the size of the sex-specific marker set. This resampling process was repeated 1,000 times, and the resulting mapping rates were used to generate the expected distribution for comparison.

### Differentiation between X and Y chromosomes

To evaluate sex-linked genomic differentiation, we calculated the *Fst* between males and females [55]. Theoretically, when X and Y chromosomes are fixed for alternative alleles, the between-sex *Fst* reaches a maximum value of 1/3 [16]. We processed the ddRAD sequencing data using the *ref_map.pl* pipeline within the STACKS software suite, mapped against the *B. gargarizans* reference genome. Subsequently, we utilized the *populations* module with the --fstats and --smooth parameters flags to compute genome-wide per-locus *Fst* values and AMOVA-*Fst* statistics, stratified by sex. To characterize the distribution of differentiation signals across the genome, we applied a locally weighted scatterplot smoothing (LOESS) model (span = 0.5) to the per-locus AMOVA-*Fst* values relative to their physical positions on each chromosome (Chr1-Chr11). The resulting trends were visualized using the *ggplot2* package. To identify candidate sex-linked regions, we established a high-differentiation threshold defined as 1.75 times the genome-wide mean of the LOESS-smoothed AMOVA-*Fst* values (threshold = 0.0865). This threshold, which corresponds to the 94–95% of the genome-wide distribution, was selected to balance statistical stringency with biological relevance [56]. Genomic regions were classified as sex-linked if their LOESS-fitted values consistently exceeded this threshold. In addition, we re‑delineated the region using thresholds of 1.5×–2.5× the mean and the 90th–99th percentiles of divergence.

### Detection of sex chromosome turnover within the *Bufo*

Leveraging the abundance of sex-linked RAD tags derived from our ddRAD-seq data, we investigated their distribution across sex chromosomes in closely related species to identify potential sex chromosome turnover events within the *Bufo* genus. We established a local reference database using the *Bufo bufo* genome assembly (GCF_905171765.1) [57] and performed sequence alignments using BLASTn (v2.6.0+). Sex-linked RAD tags were aligned against this database, with alignments retained only if they met an E-value threshold of ≤ 1 × 10⁻²⁰. To ensure alignment specificity, for loci yielding multiple hits, we required the best match to have an E-value at least five orders of magnitude lower than the second-best match. Loci failing to align or exhibiting multiple top-tier hits were documented for further exclusion or analysis.

Following the recent completion of high-quality whole-genome assemblies for *B. gargarizans* and *B. bufo* [54, 57], we performed a genome-wide synteny analysis to investigate their genomic architecture. This analysis was conducted using the ‘One Step MCScanX-Super Fast’ plugin within TBtools-II [58]. Genomic sequences (FASTA) and their corresponding annotation files (GFF3) for both species were processed according to the software’s standard requirements. The plugin was executed with default parameters to identify collinear blocks, and the resulting syntenic relationships were visualized using the ‘Dual Synteny Plot’ plugin.

## Results

### Sequencing and assembly of the ddRAD-seq

To identify sex-linked markers in *B. gargarizans*, we constructed ddRADseq libraries for 12 individuals (6 males and 6 females) (Figure S2). Sequencing yielded a total of 330,222,662 reads of raw data, with data per sample ranging from 21,394,248 to 39,667,082 (Mean = 27,518,555, SD = 5,474,677)(Table S1). Following the removal of adapter sequences, poly-N reads, and low-quality reads, 323,430,450 reads of clean data were retained, with data per sample ranging from 20,976,260 to 38,849,620 (Mean = 26,952,538, SD = 5,401,861)(Table S1). Subsequently, the clean data were demultiplexed and filtered using the *process_radtags* module within the STACKS pipeline to exclude low-quality sequences and reads with missing or ambiguous barcodes. This rigorous filtering retained 323,387,052 reads in total, with the number of reads per sample ranging from 20,973,451 to 38,844,475 (Mean = 26,948,921, SD = 5,401,161)(Figure S3). The processed data were then utilized for downstream assembly and analysis.

We performed *de novo* assembly of the filtered reads using the *denovo_map.pl* pipeline within the STACKS (parameters: M = 2, *n* = 1, m = 2), which yielded a catalog comprising 11,720,949 tags. Subsequently, the *populations* module was employed to analyze the female and male cohorts separately. Following quality control, 11,646,856 loci were retained for downstream analysis, among which 2,184,968 were identified as polymorphic variant sites.

### Identifying sex-linked markers and PCR validation

We utilized three complementary methods—Frequency (i), Heterozygosity (ii), and Sex-specific (iii)—to screen for sex-linked markers. While initial results identified candidates for both XY and ZW systems, the prevalence of XY-consistent markers was notably higher (Table 1). To mitigate false positives, we applied a two-stage filtering process: validation against opposite-sex ddRAD data followed by alignment to the *B. gargarizans* female reference genome. This stringent filtering eliminated all ZW-consistent markers as false positives. Ultimately, 256 RAD tags, containing 290 SNPs, were validated as XY-linked (i&ii: 245; iii: 11; Table 1).

**Fig. 1:**
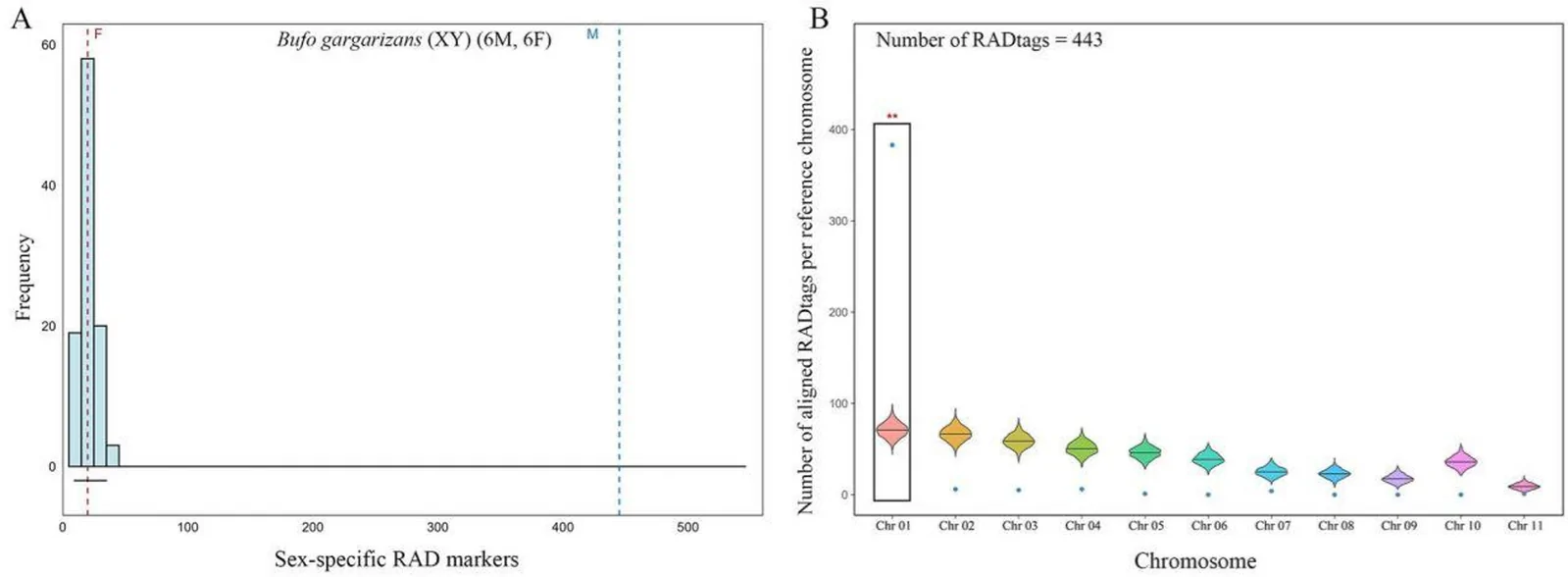
Permutation test for the distribution of sex-linked markers in the *B. gargarizans* RAD-seq dataset. (**A**) Null distribution of sex-linked marker counts generated by random permutations. Vertical blue and red lines represent the observed counts of male- and female-specific RAD markers, respectively (Table 1). Significance was determined by comparing observed values against the 95% confidence interval of the null distribution (dashed line). (**B**) Chromosomal distribution of sex-linked markers based on the reference genome. Violin plots show the distribution of markers mapped to each chromosome across 1,000 random subsamples of RAD tags (N = number of putative sex-linked markers). Blue dots indicate the observed number of sex-linked markers per chromosome. Red asterisks denote chromosomes where the observed count significantly exceeded the 99th percentile of the null distribution (*P* < 0.01)

To validate the reliability of the identified sex-linked markers, we employed a computational permutation approach. We randomly permuted sex assignments across samples and re-identified sex-linked RAD-tags (SNPs) for each iteration to determine whether the observed counts deviated significantly from random expectations. In the absence of a reference genome, we performed 100 permutations; the results demonstrated that the number of putative sex-linked markers in *B. gargarizans* significantly exceeded the null hypothesis distribution (outside the 95% confidence interval, Fig. 1A). When a reference genome was available, we conducted 1,000 random subsamplings of RAD-tags. The analysis revealed that the density of sex-linked markers on chromosome 1 was significantly higher than that expected by chance (Fig. 1B), providing robust evidence that chromosome 1 functions as the sex chromosome in *B. gargarizans*.

**Fig. 2:**
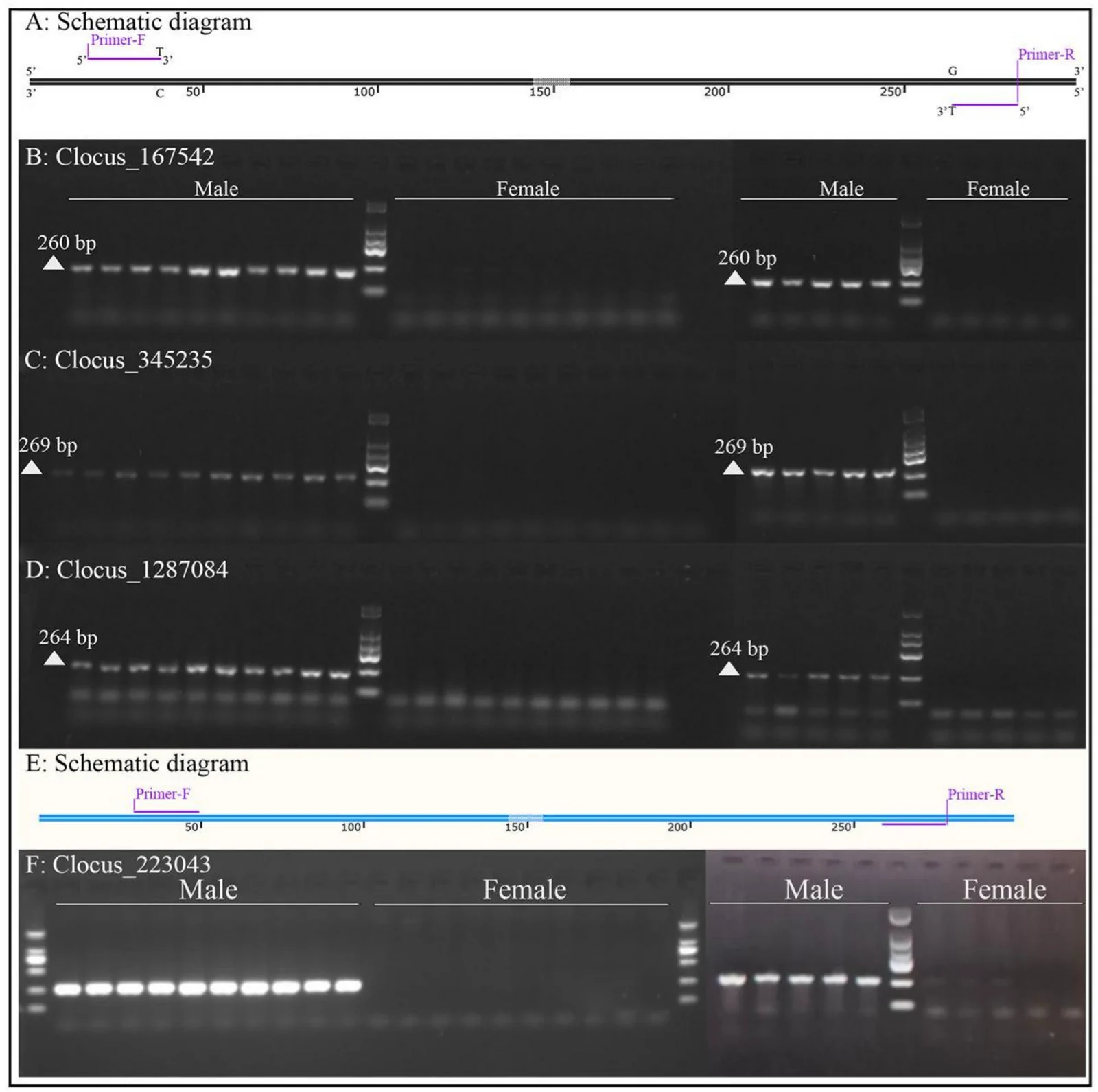
Validation of male-specific markers in *B. gargarizans. *(**A**) Schematic representation of the primer design strategy for male-specific SNP markers. (**B**–**D**) PCR validation of SNP markers for Clocus_167572 (**B**), Clocus_345235 (**C**), and Clocus_1287084 (**D**) across 15 male B. gargarizans individuals. (**E**) Schematic representation of the primer design for the male-specific Indel marker. (**F**) PCR validation of the Indel marker Clocus_223043 in 15 male individuals. Sample IDs for each lane are detailed in Table S2

**Fig. 3:**
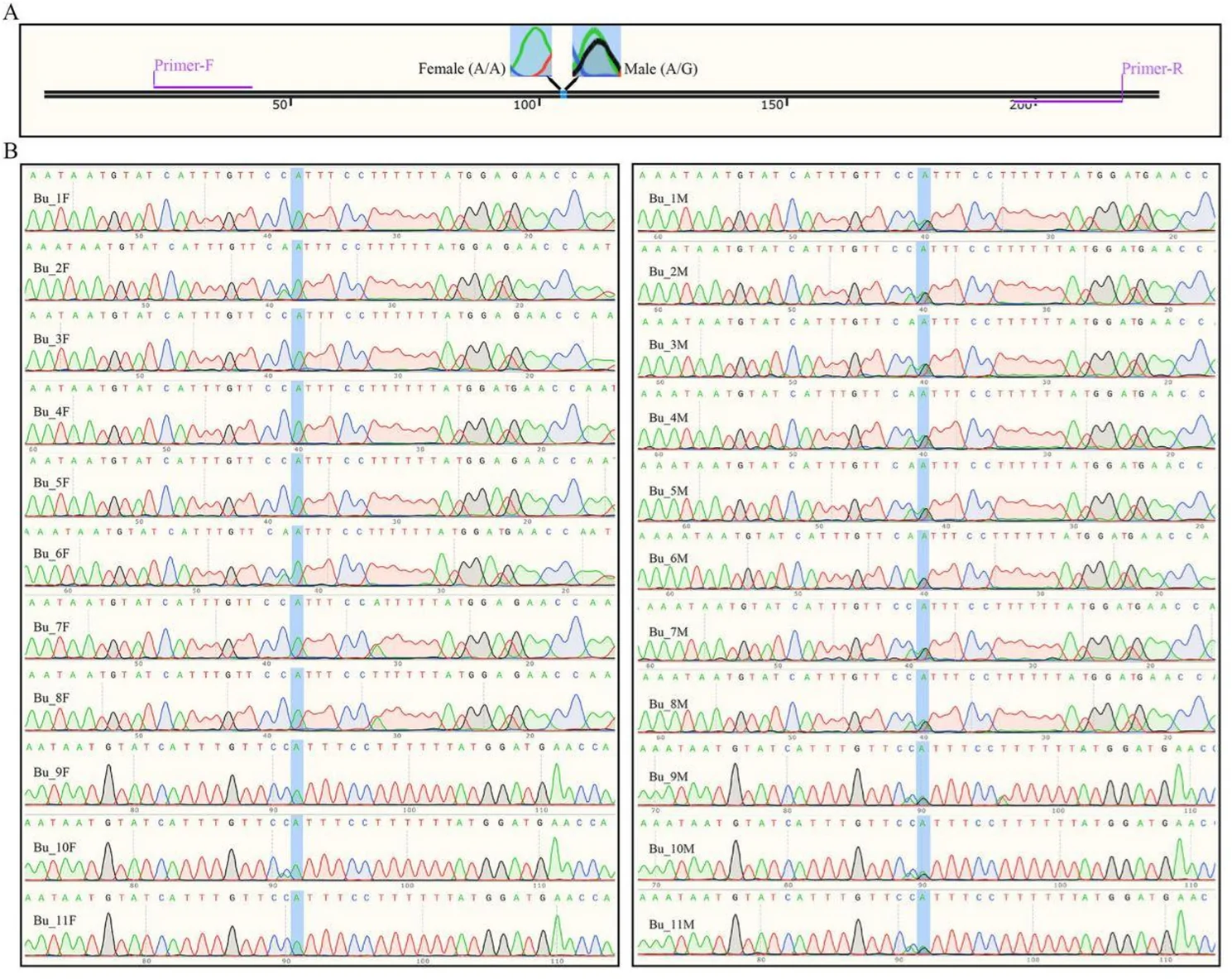
Primer design and validation of male-specific SNP markers in *B. gargarizans* using PCR-based Sanger sequencing. (**A**) Schematic overview of the validation strategy. (**B**) Sanger sequencing results demonstrating the validation of candidate markers. Primers were designed within conserved flanking regions. The target SNP sites (highlighted in blue) exhibit heterozygosity in males and homozygosity in females, confirming their sex-linked nature. Further details on the sequenced samples are available in Table S2

PCR amplification was employed to validate the sex-linked markers identified via ddRAD-seq in *B. gargarizans*. To ensure the robustness of our findings, we selected individuals independent of the initial ddRAD-seq library construction for validation (Table S2). In Test 1, the presence or absence of PCR amplicons was assessed via agarose gel electrophoresis. The sex-linked SNPs Clocus_167572, Clocus_345235, and Clocus_1287084, as well as the Indel marker Clocus_223043, consistently amplified in all 15 males but were absent in all 15 females (Fig. 2A-F). In Test 2, Sanger sequencing was performed to further confirm marker authenticity. For Clocus_23110, sequencing chromatograms revealed consistent heterozygosity (double peaks) at the SNP site in all 11 males, whereas all 11 females exhibited homozygosity (single peaks) (Fig. 3A-B). These results, corroborated by independent lines of evidence (Figure S4-S9), robustly support the conclusion that *B. gargarizans* possesses an XY heterogametic sex-determination system. All primers used in this study are listed in Supplementary Table S3.

### Identification of the *B. gargarizans* sex chromosomes

**Fig. 4:**
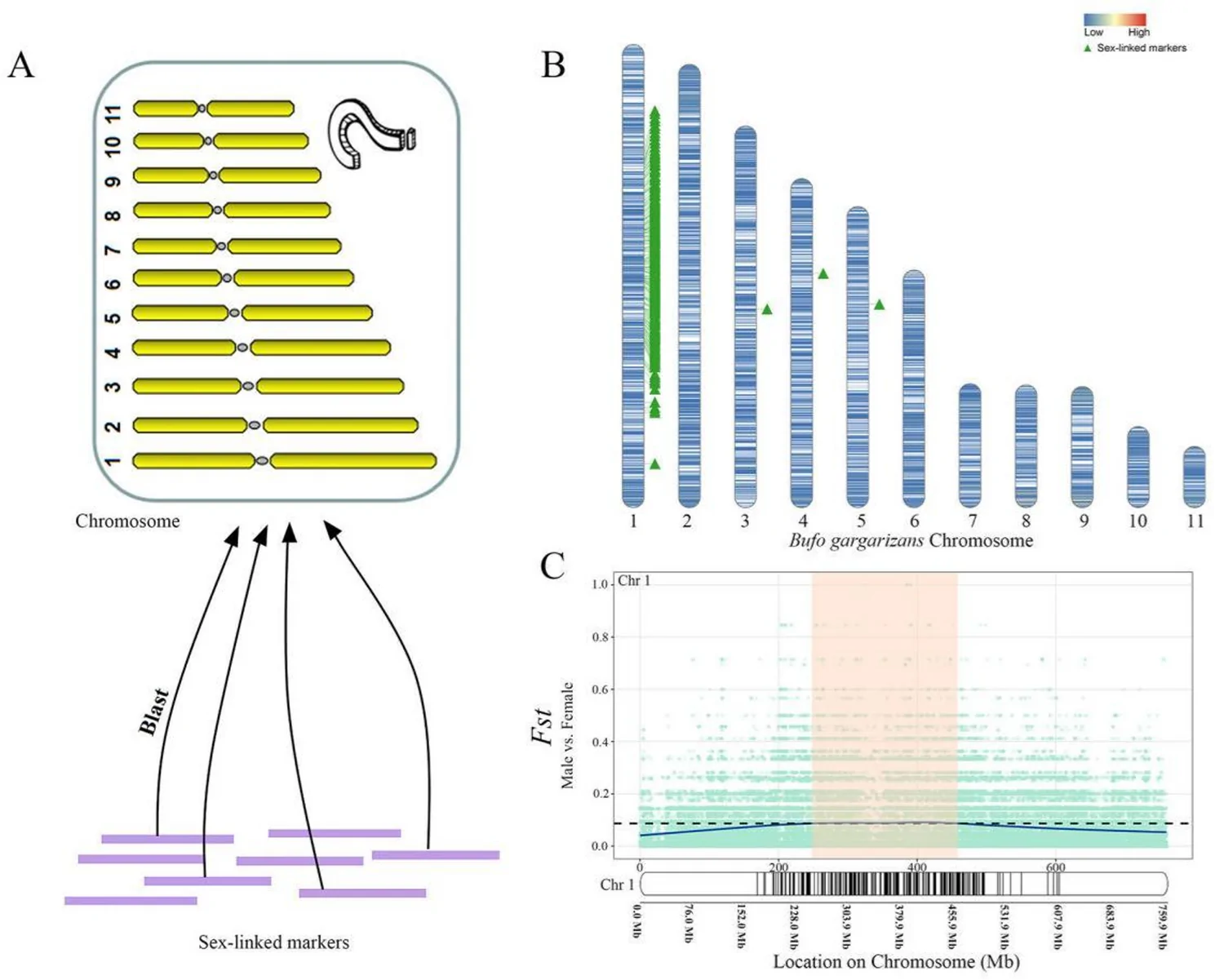
Genomic distribution of sex-linked RAD-tags and identification of the sex-linked region in *B. gargarizans. *(**A**) Schematic representation of the chromosomal distribution of sex-linked RAD-tags mapped to the B. gargarizans reference genome. (**B**) Distribution of the 245 sex-linked RAD-tags, showing that 236 (96.3%) are clustered on chromosome 1. (**C**) Identification of the putative non-recombining sex-linked region. The region spanning 246.69–458.35 Mb on chromosome 1 exhibits significantly elevated Fst values

To identify the sex chromosomes in *B. gargarizans*, we mapped the validated sex-linked RAD-tags onto the reference genome (Fig. 4A). Our analysis revealed that 236 out of 245 (96.3%) sex-linked markers were localized to chromosome 1 (Fig. 4B). Notably, these markers were clustered within the 152.0-607.9 Mb region (Fig. 4C). Combined with the permutation test results (Fig. 1B), these findings provide robust evidence that chromosome 1 functions as the sex chromosome in *B. gargarizans*, with the differentiated sex-linked segment spanning the 152.0-607.9 Mb interval.

### *Fst* values between males and females in *B. gargarizans*

**Fig. 5:**
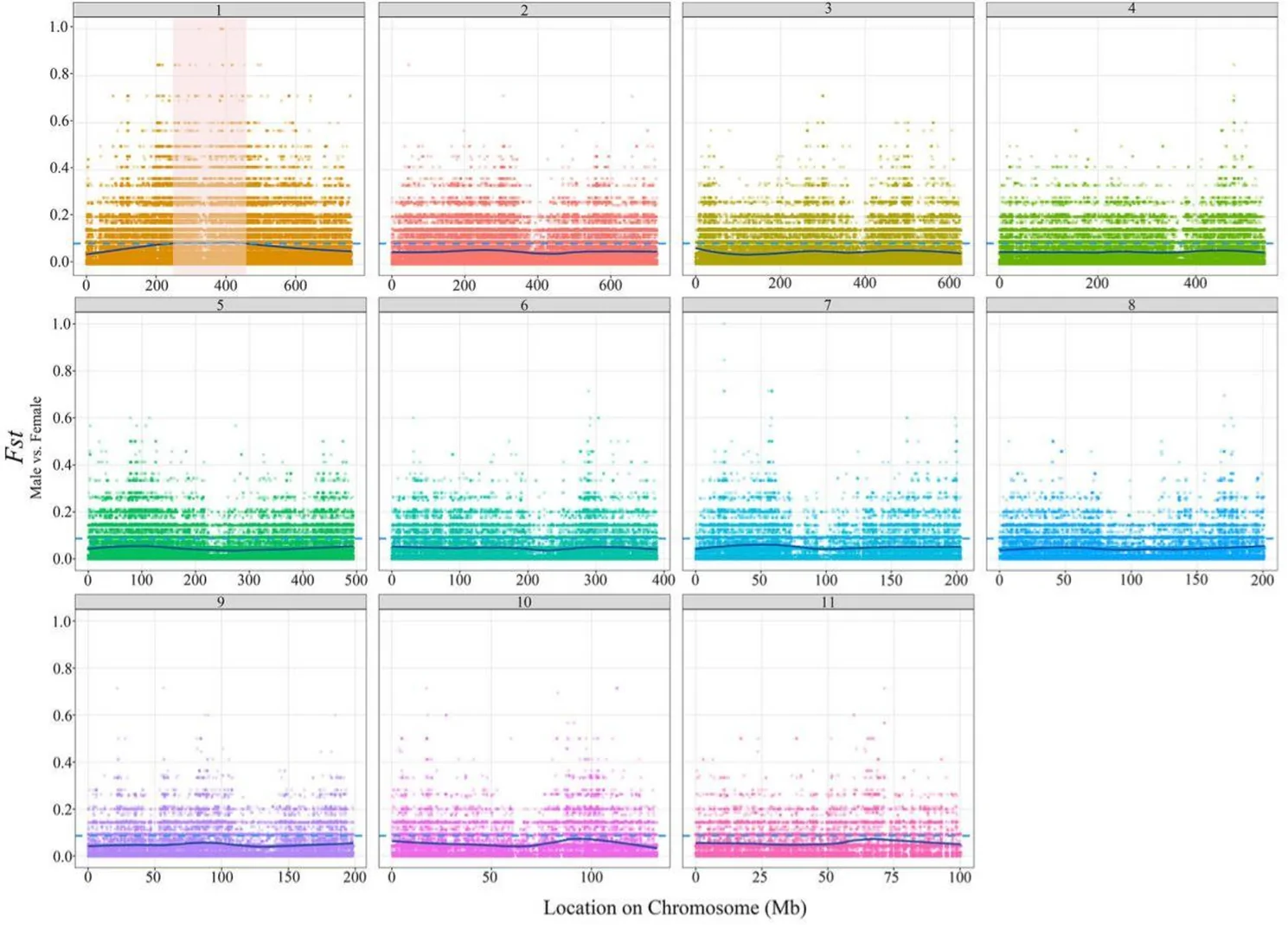
Genome-wide *Fst* distribution between male and female *B. gargarizans. *Chromosome-specific Fst values were calculated by comparing male and female populations, using the female B. gargarizans genome as the reference assembly. Each dot represents the Fst value of an individual locus. The blue line indicates the trend estimated via Loess regression, and the shaded areas highlight genomic regions exhibiting high differentiation

To quantify the differentiation between the X and Y chromosomes, we calculated *Fst* values, a robust metric for detecting divergence in sex chromosomes, particularly those exhibiting low levels of differentiation. Our analysis revealed that chromosome 1 of *B. gargarizans* displays characteristics typical of sex-linked inheritance. Specifically, *Fst* values between males and females were significantly elevated within the 246.69-458.35 Mb region, identifying a 212 Mb segment of pronounced chromosomal differentiation (Fig. 4C). The gradual yet distinct decline in *Fst* values flanking this peak suggests a low but non-zero recombination rate between the X and Y chromosomes. No significant intersexual differentiation was detected on any other chromosomes (Fig. 5). In addition, among the mean‑based thresholds, the 2.0× and 2.5× cutoffs yielded no detectable NRR because they exceeded the actual peak of the LOESS curve on Chr1 (~ 0.091); this does not undermine our conclusions, but rather confirms that our chosen threshold (0.0865) lies within a plausible range for capturing the core NRR. Both the 90th and 95th percentile thresholds recapitulated the NRR defined by the original 0.0865 cutoff. Although the 99th percentile produced a more fragmented pattern, the core high‑divergence region remained entirely within the original NRR (Figure S12-S15).

### Detection of sex chromosome turnover within the *Bufo*

Given the close phylogenetic relationship between *B. gargarizans* and *B. bufo*, we investigated whether intragenus sex chromosome turnover has occurred between these two species. To test this, we mapped the sex-linked RAD-tags (SNPs) identified in *B. gargarizans* against the reference genome of *B. bufo* using BLASTn. Our analysis revealed that 194 of the 245 sex-linked RAD-tags (79.2%) aligned to chromosome 2 (Chr2) of *B. bufo*. This substantial synteny suggests that the sex-linked region on Chr1 in *B. gargarizans* is highly homologous to the corresponding region on Chr2 in *B. bufo*, providing evidence for conserved chromosomal blocks but divergent chromosome identities or potential chromosomal rearrangements between these closely related species.

**Fig. 6:**
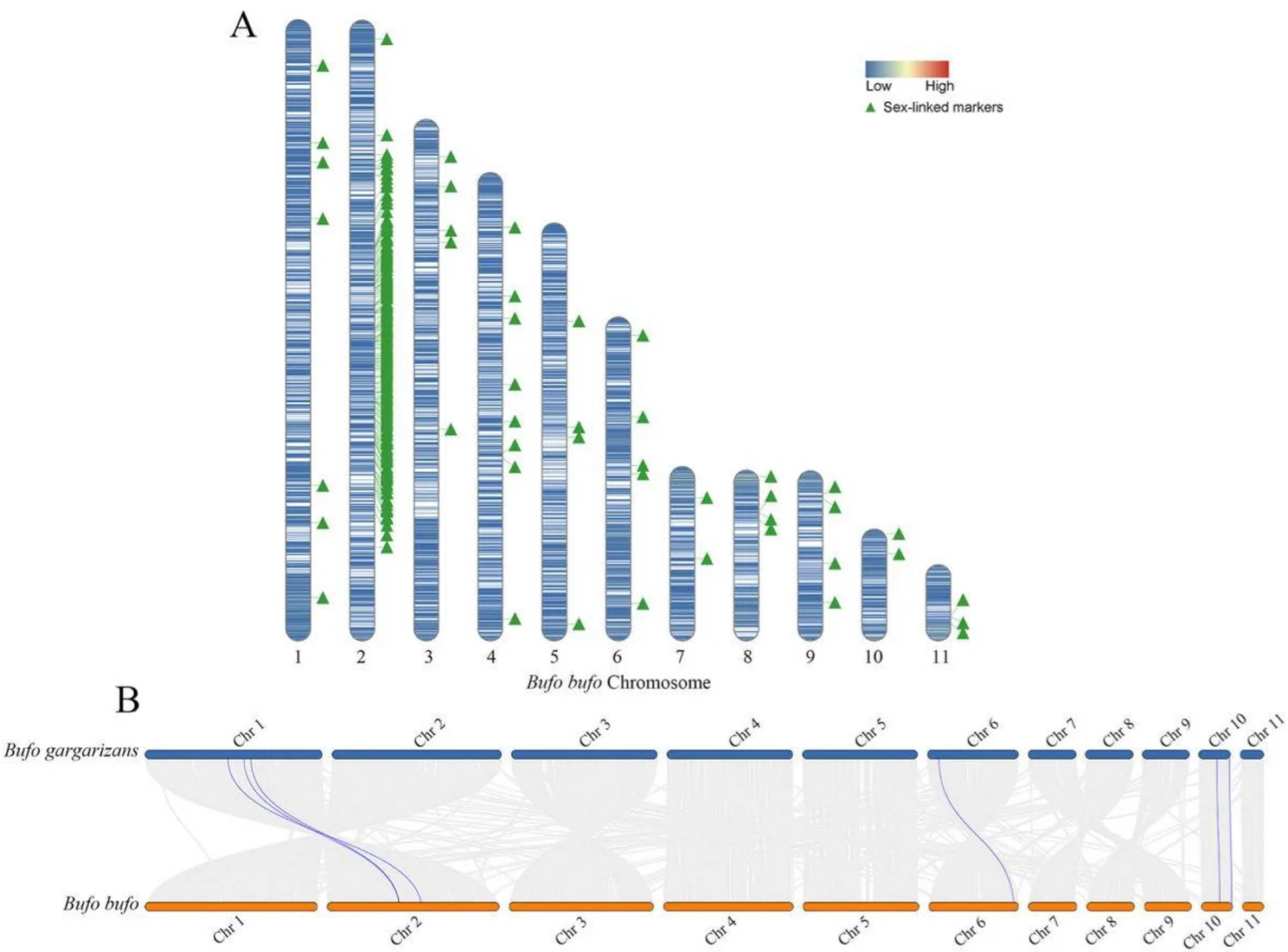
Genomic mapping of sex-linked RAD-tags and synteny analysis between *B. gargarizans* and *B. bufo. *(**A**)Distribution of 245 sex-linked RAD-tags (SNPs) mapped to the B. bufo reference genome, with 194 SNPs localized to chromosome 2. (**B**) Synteny map between the chromosome-level assemblies of B. gargarizans and B. bufo. Blue bands highlight orthologous genes associated with sex determination, differentiation, or gonadal development. Note the chromosomal locations of key genes: DMRT1 and AMH are located on chromosome 1 of B. gargarizans and chromosome 2 of B. bufo, whereas SOX9, SF1, and WT1 are syntenic on chromosome 6 in both species

Recent whole-genome assemblies for *B. gargarizans* and *B. bufo* have facilitated a comprehensive synteny analysis between these two species [54, 56]. Our results demonstrate a high degree of genomic conservation; however, we identified notable discrepancies in chromosome nomenclature and orientation. Specifically, several chromosomes exhibit non-correspondence in numbering (e.g., *B. gargarizans* Chr1 vs. *B. bufo* Chr2), a finding corroborated by sex-linked RAD-tag mapping. Furthermore, multiple chromosomal rearrangements, particularly inversion events, were detected across several homologous pairs (e.g., Chr1/Chr2, Chr2/Chr1, and Chr3/Chr3; see Fig. 6 for detailed correspondences). These structural variations highlight the evolutionary divergence between the two lineages despite their overall syntenic conservation.

Our previous findings identified Chr1 as the sex chromosome in *B. gargarizans*. Whole-genome synteny analysis revealed a high degree of homology between *B. gargarizans* Chr1 and *B. bufo* Chr2. While this homology might initially suggest that *B. bufo* Chr2 serves as a sex chromosome, established literature indicates that *B. bufo* utilizes a ZW sex-determination system, with sex-linkage mapped to Chr6 [59]. By integrating these findings, we conclude that a non-homologous sex chromosome turnover has occurred within the *Bufo* genus: the XY system in *B. gargarizans* is localized to Chr1, whereas the ZW system in *B. bufo* resides on Chr6.

## Discussion

ddRAD-seq is a cost-effective Reduced Representation Sequencing (RRS) technique widely utilized for genomic studies in non-model organisms. By employing two restriction enzymes to selectively sequence genomic fragments, this approach substantially reduces genomic complexity, facilitating high-density SNP discovery [60, 61]. In this study, we applied ddRAD-seq, integrated with rigorous bioinformatic filtering and experimental validation, to characterize sex-linked markers in *B. gargarizans*. Our results provide the first evidence of an XY sex-determination system in this species, with the sex-determining region localized to Chr1. These findings not only elucidate the genetic basis of sex determination in *B. gargarizans* but also offer novel insights into the evolutionary dynamics of sex chromosomes within the genus *Bufo* through comparative genomic analysis. Finally, all 30 independent individuals used for validation were sampled from a single geographic region (Quzhou City, Zhejiang Province, China). Given population genetic evidence suggesting that sex determination mechanisms—particularly in homomorphic sex chromosome systems—can vary geographically, this reliance on a single population source may limit the generalizability of our conclusions. In future work, we plan to employ larger sample sizes drawn from a broader geographic range to further validate the universality of the XX/XY system.

### The XY sex determination system and sex chromosomes in *B. gargarizans*

Our analysis provides robust evidence that sex determination in *B. gargarizans* is governed by an XY heterogametic system. This conclusion is supported by a tripartite validation strategy. Initially, we identified candidate sex-linked markers using three complementary approaches—the Frequency, Heterozygosity, and Sex-specific methods—retaining only those that conformed to the expected XY inheritance patterns after rigorous filtering of false positives (Table 1). Subsequently, permutation tests demonstrated that the observed frequency of male-specific markers significantly exceeded random expectations (Fig. 1A), effectively ruling out stochastic occurrence. Finally, experimental validation via PCR amplification and sequencing confirmed the predicted segregation patterns: markers were either male-specific or heterozygous in males while absent or homozygous in females (Figs. 2 and 3). These findings are consistent with the theoretical framework of an XY sex-determination system, wherein Y-linked markers are exclusively associated with the male genotype.

For *B. gargarizans*, we precisely mapped the sex-linked markers to chromosome 1, confirming its functional role as the sex chromosome in this species [59, 62]. Dufresnes et al. (2025) reported that 84 out of 104 sex-linked RAD-tags (SNPs) in *B. spinosus* localized to a large region of the *B. bufo* Chr 2 (233–623 Mb), identifying it as the sex chromosome in that species [59]. In our study, 194 out of 245 sex-linked RAD-tags (SNPs) in *B. gargarizans* also mapped to the *B. bufo* Chr2 (approximately 300–640 Mb; Figure S10). This high degree of synteny suggests a conserved sex-determining region between *B. gargarizans* and *B. spinosus*. While we could not directly map the sex-linked RAD-tags from *B. spinosus* onto the *B. gargarizans* reference genome, the observed genomic distribution patterns lead us to hypothesize that Chr 1 in *B. gargarizans* may share an ancestral chromosomal origin with *B. spinosus*, yet have followed divergent evolutionary trajectories as sex chromosomes in different lineages.

### Revealing XY chromosome divergence with *Fst*

To investigate sex chromosome differentiation in *B. gargarizans*, we employed *Fst* analysis, which effectively captures allele frequency differences between sexes [16, 55]. We identified a significant peak of *Fst* values on Chr1, indicative of a NRR spanning approximately 212 Mb (246.69-458.35 Mb). Furthermore, we acknowledge that the 212 Mb NRR boundary, derived from our discovery dataset, is a preliminary estimate and should not be regarded as a definitive, calibrated population‑level conclusion. This will be refined in subsequent studies. In many vertebrate systems, recombination suppression is closely linked to Y/W chromosome degeneration and subsequent gene loss [9–13, 63], as exemplified by the well-characterized sex chromosome systems in mammals [64, 65] and birds [66]. However, *B. gargarizans* presents a notable exception. Despite the presence of a large NRR, the sex chromosomes remain morphologically indistinguishable. This configuration aligns with emerging evidence from *Rana temporaria* [16, 67]and *Leptobrachium boringii* [68], suggesting that extensive sequence divergence can evolve on sex chromosomes without triggering the rapid degeneration typically associated with suppressed recombination.

### Sex chromosome turnover within *Bufo*

Amphibians are characterized by rapid sex chromosome turnover, with diverse sex-determination systems frequently observed even among closely related species or distinct geographic populations [14, 69]. A striking example is the genus *Bufo*, where *B. bufo* exhibits a ZW system (female heterogamety), whereas its sister species *B. spinosus* utilizes an XY system (male heterogamety) [59, 62]. Furthermore, *B. gargarizans* also possesses an XY system; however, its sex chromosomes are non-homologous to those of *B. bufo*, indicating a shift in sex chromosome identity within the genus. While these observations suggest a complex evolutionary history of sex chromosome turnover in *Bufo*, systematic phylogenetic tracing is required to elucidate the mechanisms underlying these transitions [59].

### Candidate sex-determining gene

Drawing on the framework established by Dufresnes et al. (2025), we mapped orthologs associated with sex determination, sexual differentiation, and gonad development onto the reference genomes of *B. gargarizans* and *B. bufo*. Our analysis revealed that key vertebrate sex-determining genes—including *DMRT1*, *DMRT2*, *DMRT3*, and *AMH*—are significantly clustered within the region exhibiting the highest density of sex-linked markers on Chr1 of *B. gargarizans* (Figure S11). This syntenic region, located on Chr2 in *B. bufo* (Figure S11), also harbors several genes implicated in spermatogenesis, such as *SPMAP2*, *SPATA6L*, and *TDRD7*. In contrast, *SOX9* was consistently mapped to Chr6 in both species, suggesting a conserved genomic location.

*DMRT1* is widely recognized as a master regulator of sex determination across various vertebrate lineages. This conserved role is exemplified by its paralogs, which have evolved specialized functions in specific taxa. For instance, in *Xenopus laevis*, the W-linked *DM-W*—a duplicated paralog of *DMRT1*—functions as a female sex-determining gene [70]. Similarly, in the genus Oryzias, the Y-linked *DMY* (also known as *Dmrt1bY*) has been identified as the primary sex-determining factor in *O. latipes* [71, 72]. Beyond its role as a master switch, *DMRT1* orchestrates downstream gonadal development through intricate signaling networks. It is known to indirectly activate *AMH*, a critical mediator of Müllerian duct regression and testis formation, via the HMG-box transcription factor *SOX9* [73, 74]. The functional significance of this pathway is underscored by the observation that SOX9 mutations in mammals result in sex reversal [75, 76]. Furthermore, *DMRT1* has been shown to directly interact with *SOX9* to synergistically promote testicular development while concurrently suppressing ovarian-promoting pathways [77].

Given the high functional conservation of *DMRT1* across vertebrates, our findings underscore its pivotal role within the genetic regulatory network governing sex determination and gonadal differentiation [78, 79]. We hypothesize that *DMRT1* functions as the master sex-determining gene in *B. gargarizans*, a proposition warranting further experimental validation. Should this hypothesis hold, the regulatory hierarchy suggests that a specific allele on Chr1 of *B. gargarizans* likely upregulates *DMRT1*, subsequently triggering the expression of *SOX9* and *AMH*—a mechanism that can be verified through detailed sequence analysis and expression profiling, although this remains speculative at present. Furthermore, if *DMRT1* on Chr1 acts as the master regulator in *B. gargarizans*, it implies that *SOX9* on Chr6 may have assumed this role in *B. bufo* through a turnover event. Collectively, these results reinforce the paradigm that evolutionary transitions in sex determination frequently occur at chromosomes harboring genes at the apex of the regulatory cascade [14, 80, 81].

## Conclusions

Our study provides robust genomic evidence that the Asian toad (*B. gargarizans*) possesses an XX/XY sex-determination system, with chromosome 1 acting as the master sex chromosome. This finding fundamentally revises long-standing cytogenetic models that had previously posited a ZZ/ZW system in this species. By integrating ddRAD-seq and population genomic analyses, we identified a substantial (~ 212 Mb) non-recombining region on chromosome 1, characterized by significant sequence divergence between the X and Y chromosomes. Crucially, our research offers new insights into the evolutionary dynamics within the genus *Bufo*. Comparative genomic analyses reveal that the XX/XY system in *B. gargarizans* contrasts sharply with the ZZ/ZW system observed in its close relative, *B. bufo*. This provides a compelling, high-resolution case study of rapid sex-chromosome turnover between closely related species.

## Supplementary Information

Supplementary Material 1.

Supplementary Material 2.

Supplementary Material 3.

Supplementary Material 4.

Supplementary Material 5.

Supplementary Material 6.

Supplementary Material 7.

## Data Availability

The ddRAD-seq data generated in this study were deposited in the NCBI database (https://www.ncbi.nlm.nih.gov/) under BioProjectID PRJNA1454695, Genome Sequence Archive (GSA) (https://ngdc.cncb.ac.cn/gsa/) under CRA042807, and Science Data Bank (DOI:10.57760/sciencedb.j00139.00296).
