# Supplementary material for: Identification of sex-linked molecular markers in the Asiatic toad (Bufo gargarizans Cantor, 1842) reveals an XX/XY sex determination system
Source: BMC Genomics. 2026 Aug 14;27:935. doi: 10.1186/s12864-026-13266-w (PMC13753293; doi:10.1186/s12864-026-13266-w)
Supplement: Supplementary file 1 [file 12864_2026_13266_MOESM1_ESM.pdf]

Supplemental Information

Identification of sex-linked molecular markers in the Asiatic toad (*Bufo gargarizans* Cantor, 1842) reveals an XX/XY sex determination system

Contents

Figure S1. 2
Figure S2. 2
Figure S3. 3
Figure S4. 4
Figure S5. 5
Figure S6. 6
Figure S7. 7
Figure S8. 8
Figure S9. 9
Figure S10. 9
Figure S11. 10

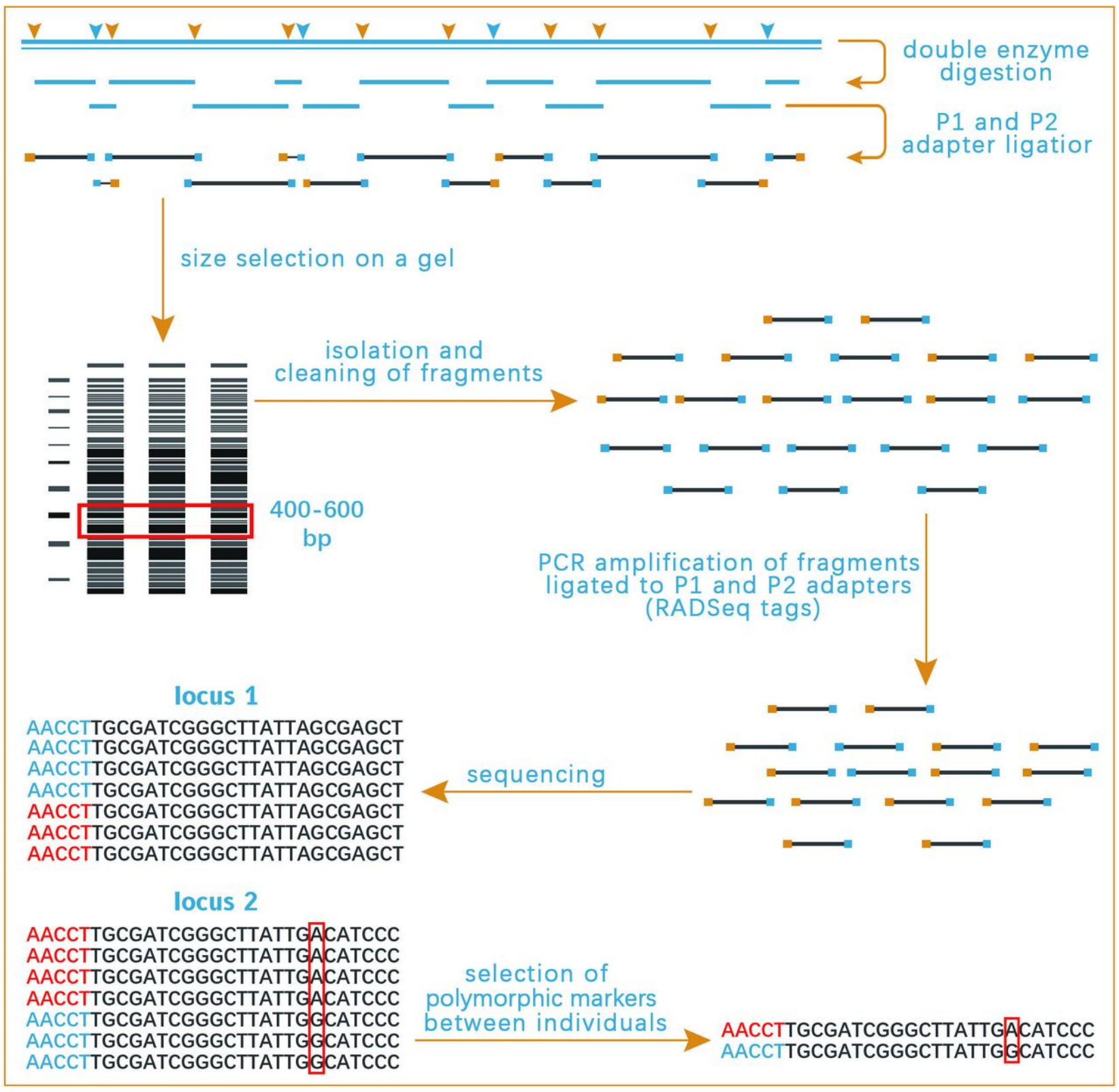

Figure S1. Schematic diagram of the ddRAD experimental workflow.

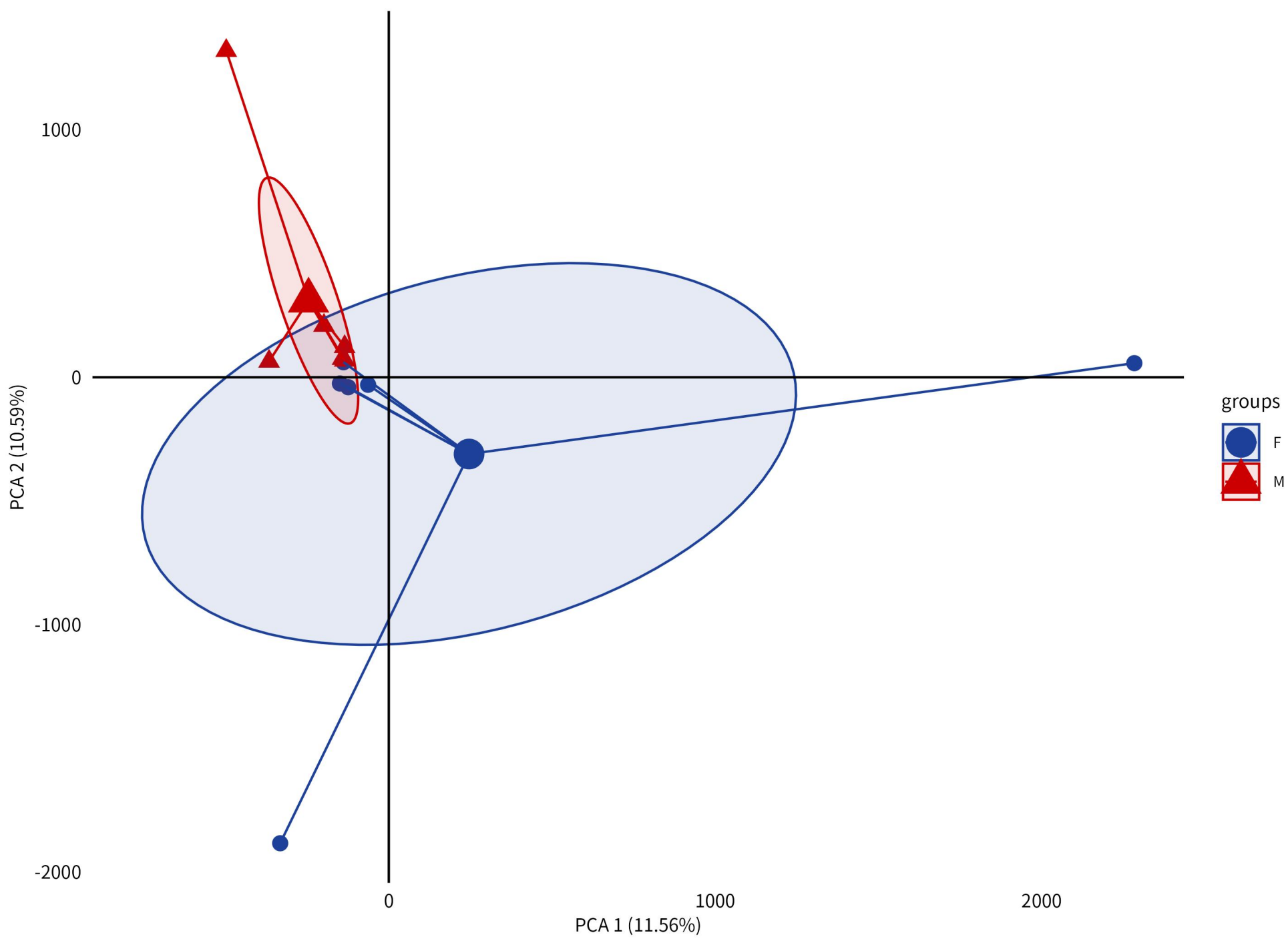

Figure S2. PCA of 12 individuals in two populations based on SNPs present in 100% samples, computed using ADEgenet.

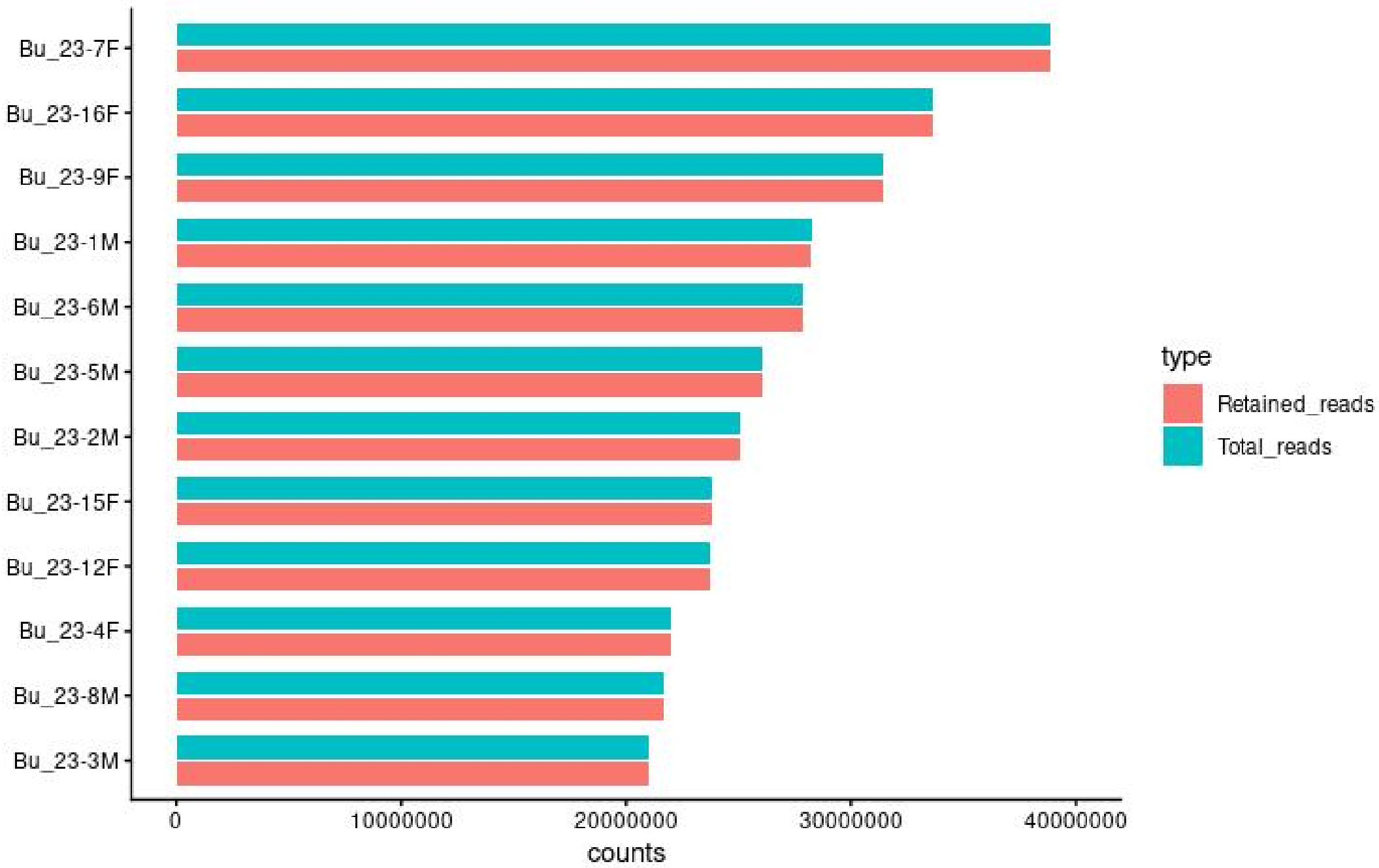

Figure S3. The results from *Process_radtags* of *B. gargarizans*.

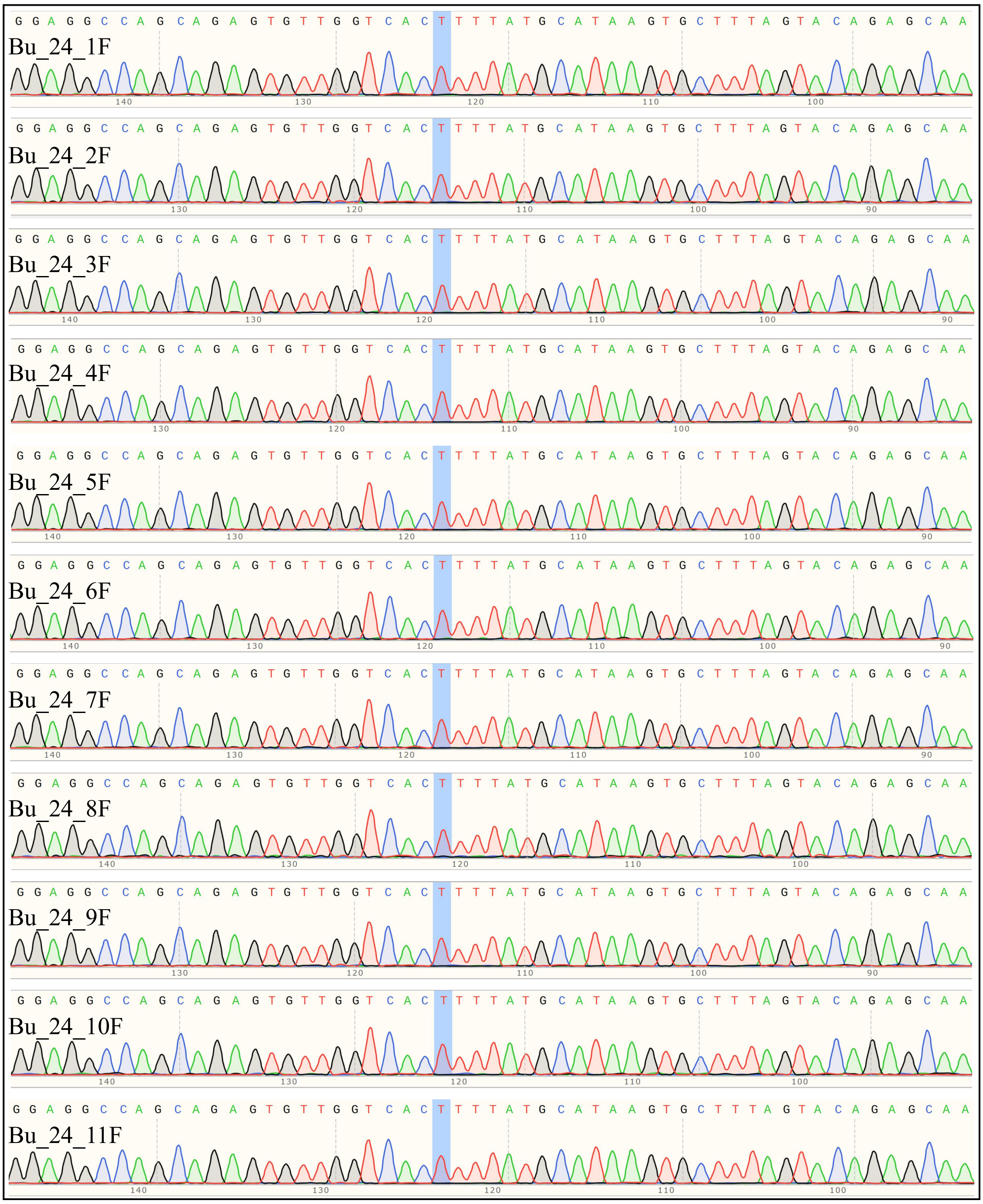

Figure S4 The PCR validation results for the sex-linked marker Clocus_183021. The variable loci were shown in blue boxes. The results indicate that female *B. gargarizans* individuals are homozygous (T/T) at this locus.

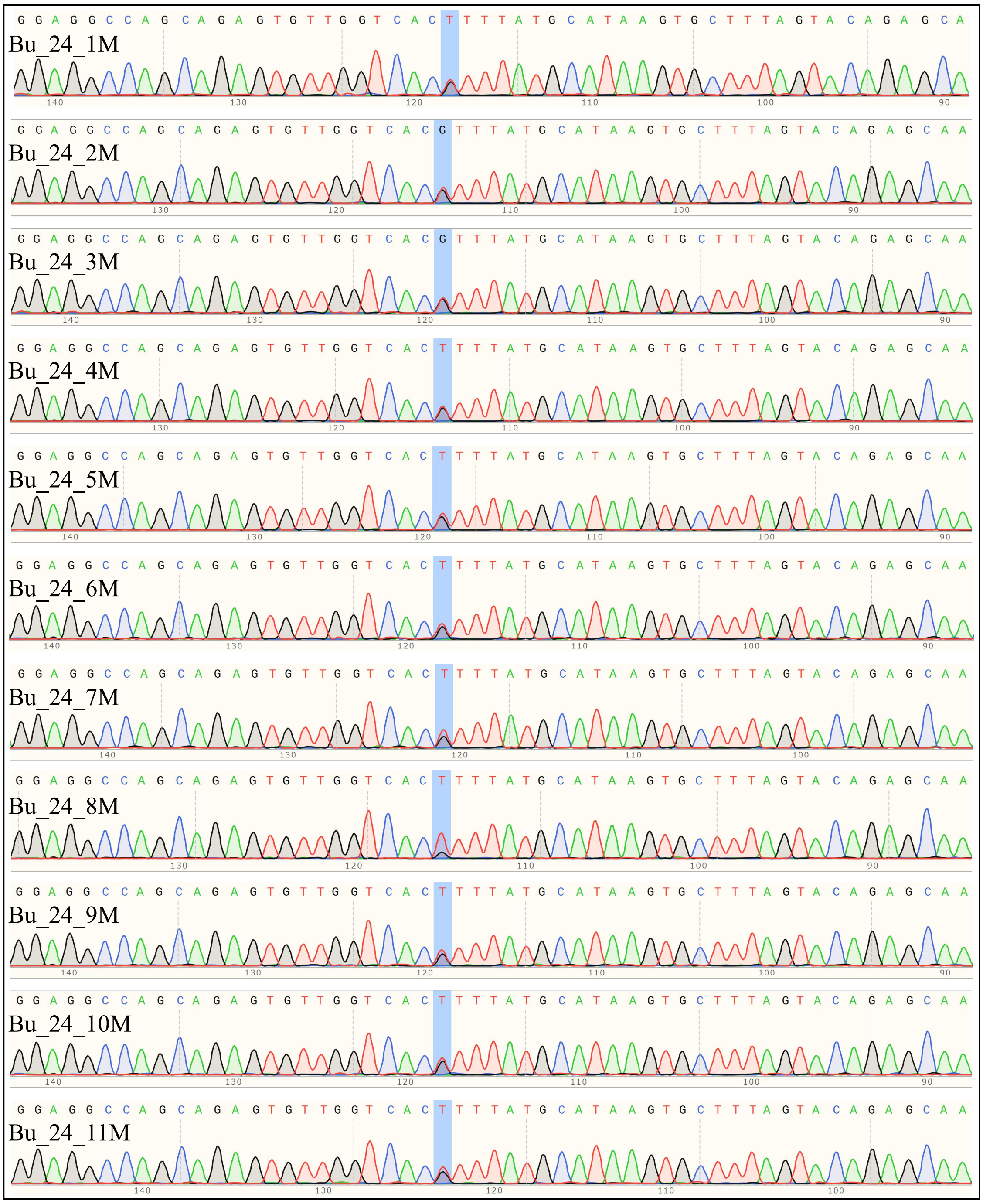

Figure S5. The PCR validation results for the sex-linked marker Clocus_183021. The results indicate that male *B. gargarizans* individuals are heterozygous (T/G) at this locus.

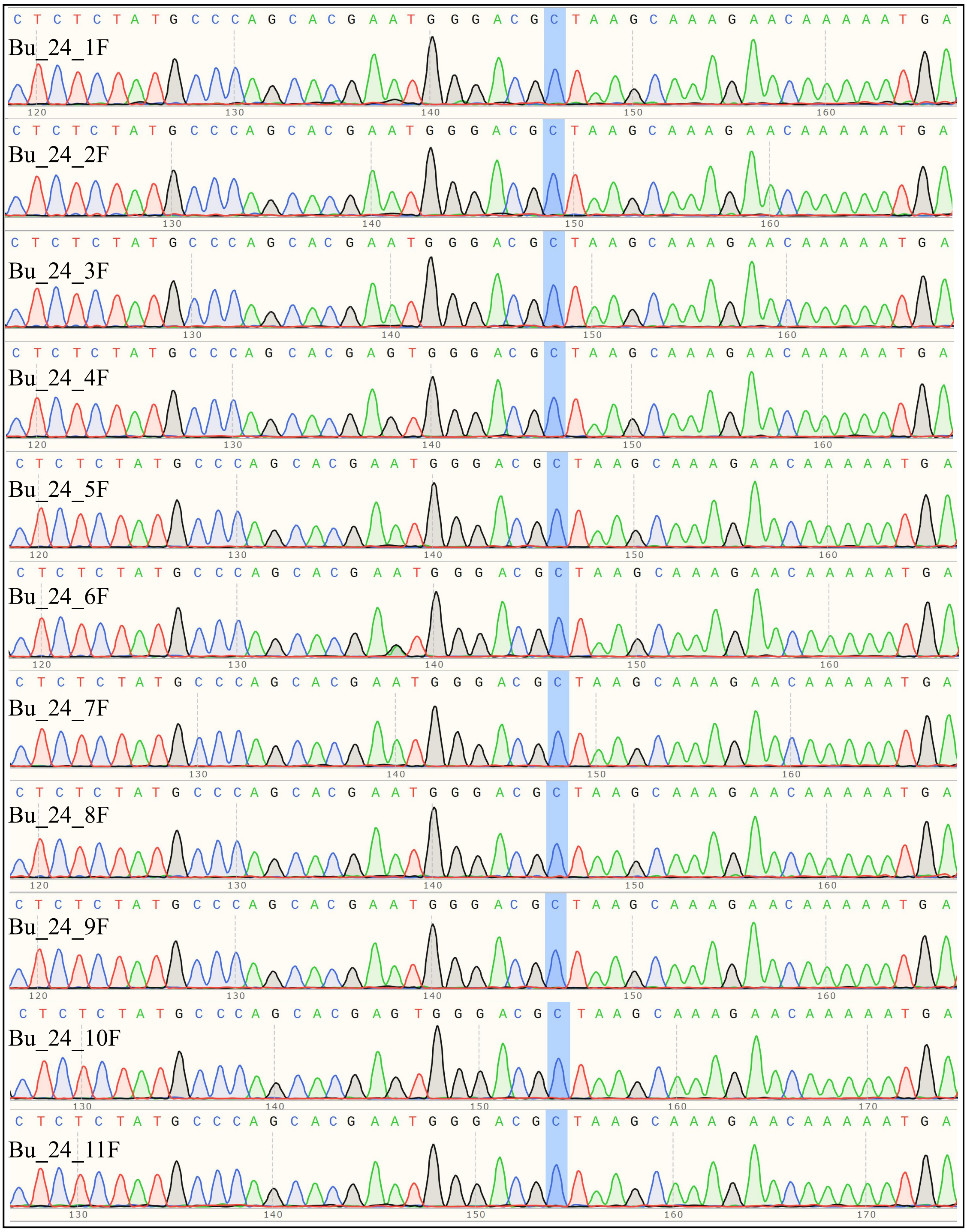

Figure S6. The PCR validation results for the sex-linked marker Clocus_715040. The results indicate that female *B. gargarizans* individuals are homozygous (C/C) at this locus.

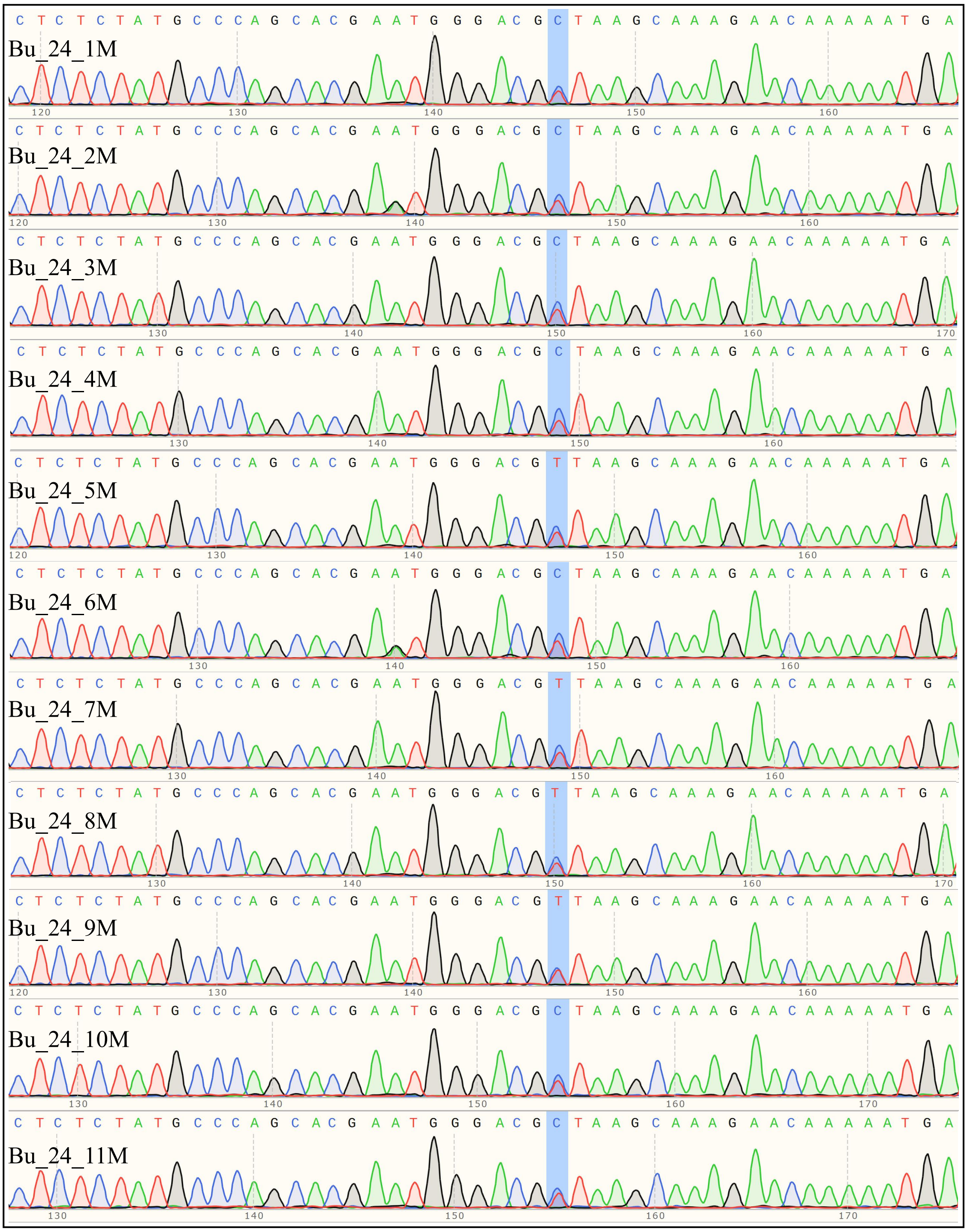

Figure S7. The PCR validation results for the sex-linked marker Clocus_715040. The results indicate that male *B. gargarizans* individuals are heterozygous (C/T) at this locus.

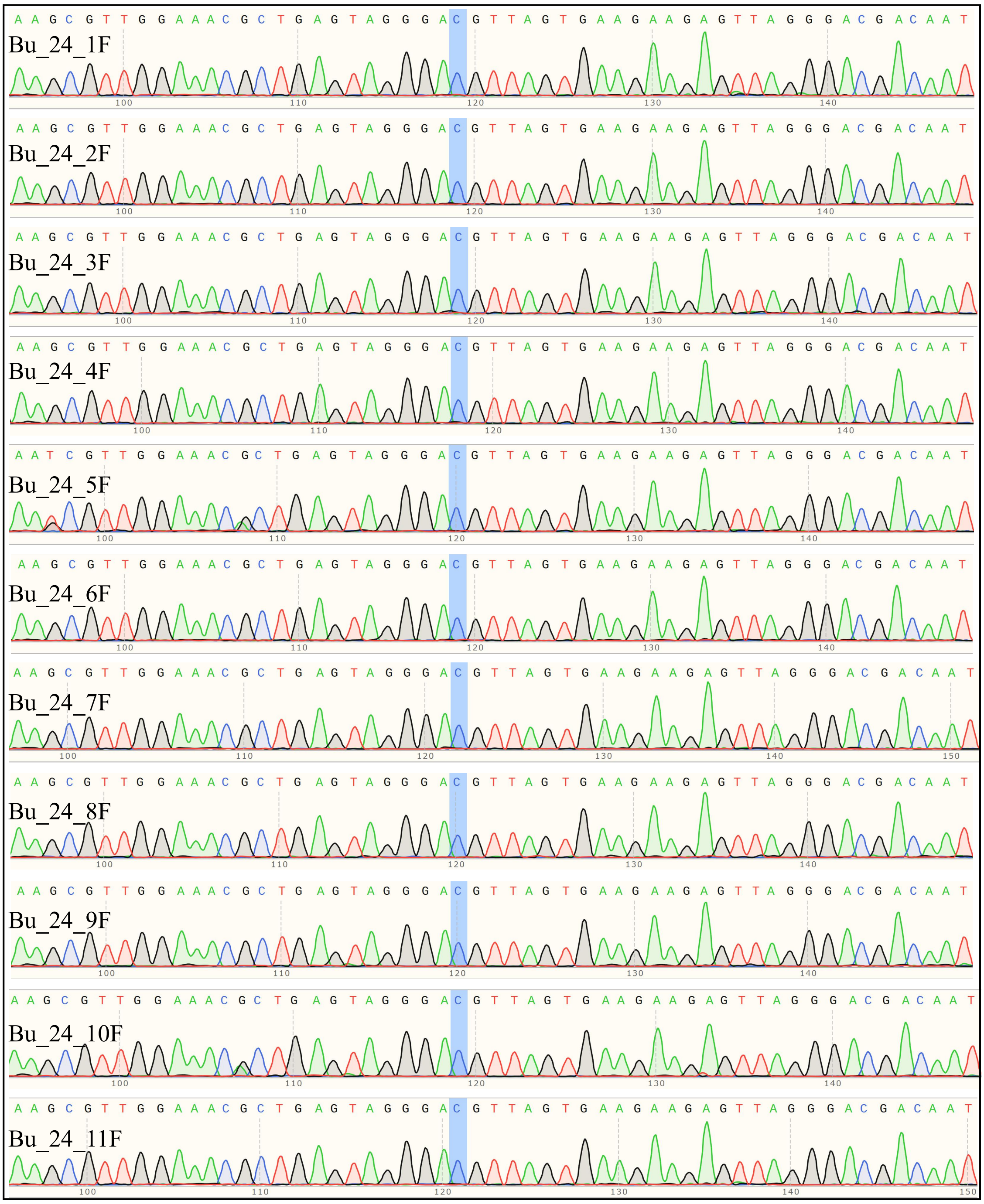

Figure S8. The PCR validation results for the sex-linked marker Clocus_1335887. The results indicate that female *B. gargarizans* individuals are homozygous (C/C) at this locus.

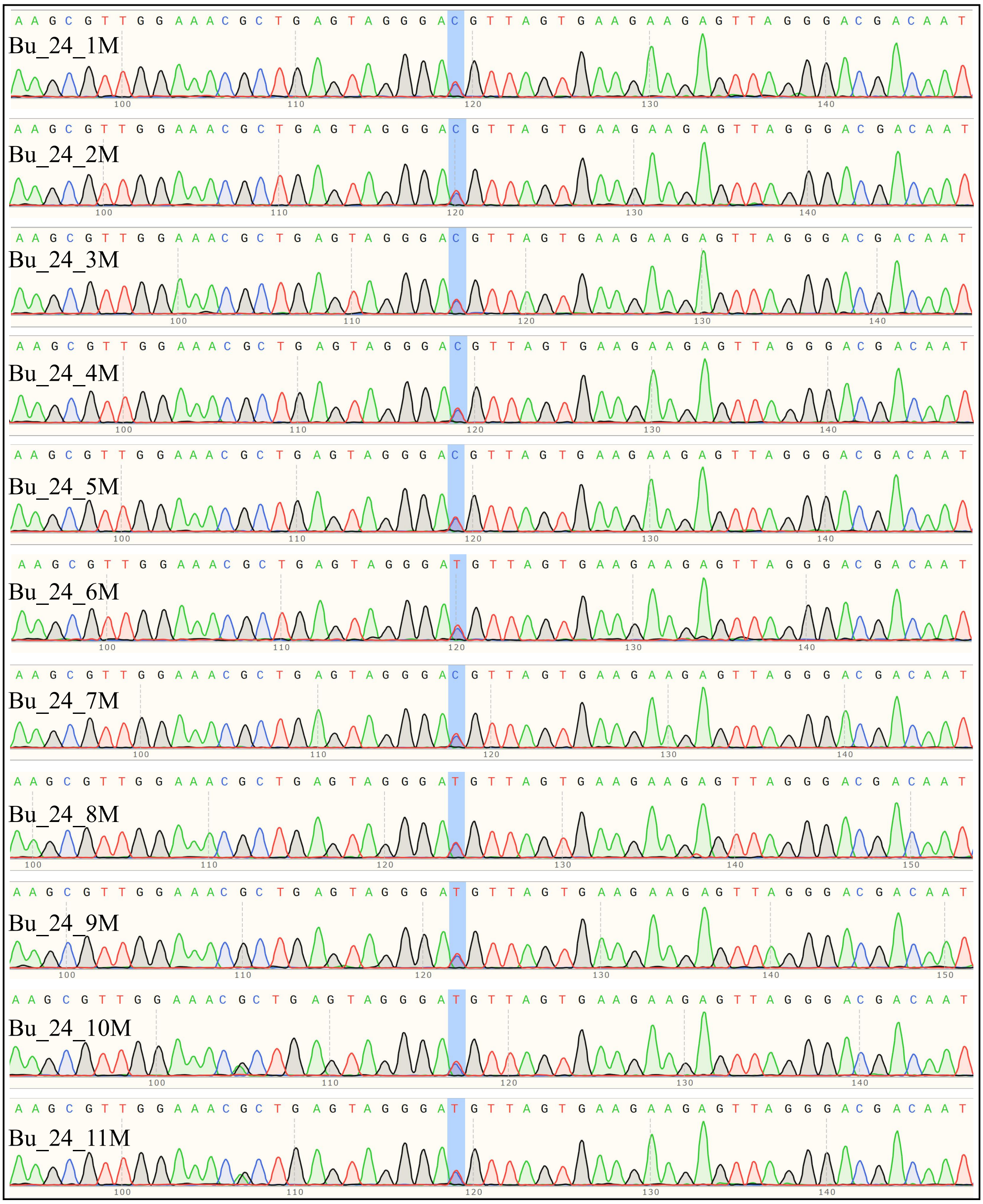

Figure S9. The PCR validation results for the sex-linked marker Clocus_1335887. The results indicate that male *B. gargarizans* individuals are heterozygous (C/T) at this locus.

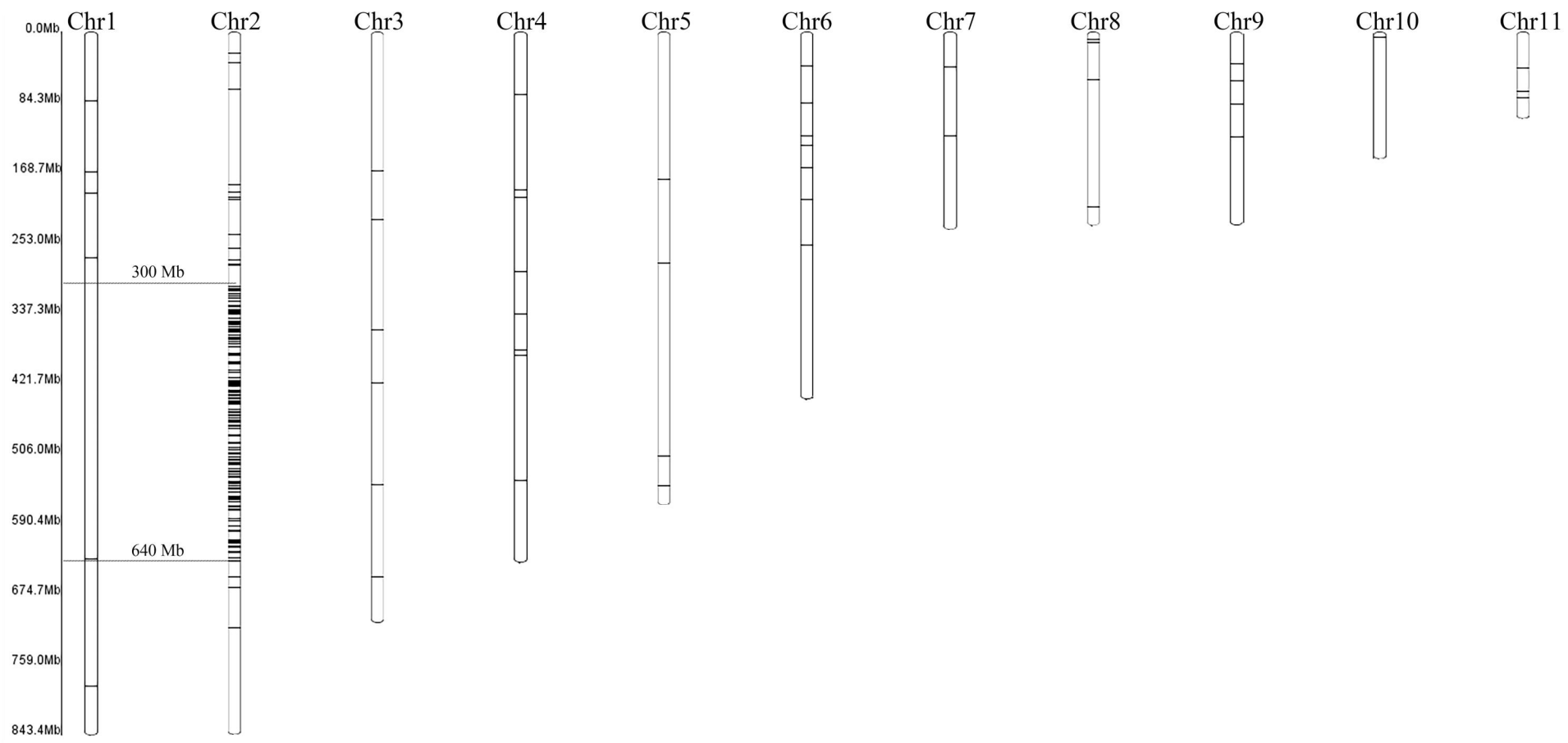

Figure S10. The patterns of sex-linked RAD-tag (SNPs) markers in *B. gargarizans* as compared to the *B. bufo* reference chromosome assembly are described. Sex-linked RAD-tag (SNP) are indicated by small black horizontal lines.

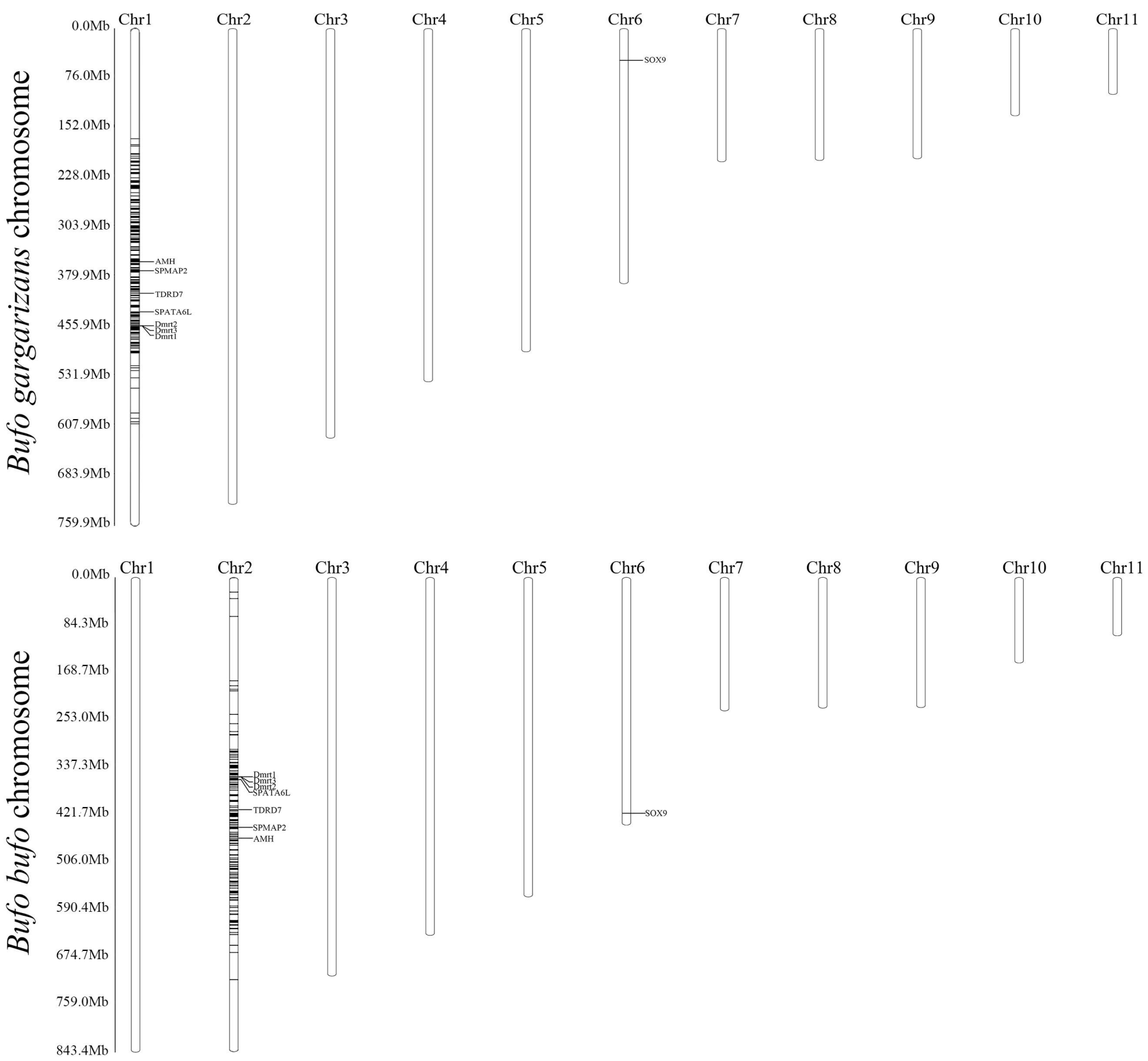

Figure S11. Known genes involved in sex determination, sexual differentiation, or gonad development were mapped onto the reference genomes of *B. gargarizans* and *B. bufo*. Sex-linked RAD-tag (SNP) are indicated by small black horizontal lines.
