# Supplementary material for: Identification of sex-linked molecular markers in the Asiatic toad (Bufo gargarizans Cantor, 1842) reveals an XX/XY sex determination system
Source: BMC Genomics. 2026 Aug 14;27:935. doi: 10.1186/s12864-026-13266-w (PMC13753293; doi:10.1186/s12864-026-13266-w)
Supplement: Supplementary file 2 [file 12864_2026_13266_MOESM2_ESM.pdf]

Table S1 Quality summary of *Bufo gargarizans* (6♂，6 ♀) by ddRAD sequencing

Serial	Sample	Raw Base(bp)	Clean Base(bp)	Effective Rate(%)	Error Rate(%)	Q20(%)	Q30(%)	GC Content(%)
1	Bu_23-1M	4,159,307,808	4,065,934,752	100.00	0.04	97.58	93.23	43.49
2	Bu_23-2M	3,696,946,272	3,613,419,648	100.00	0.04	97.61	93.29	43.38
3	Bu_23-3M	3,080,771,712	302,0581,440	100.00	0.04	97.43	92.89	43.56
4	Bu_23-5M	3,856,622,976	3,745,880,640	100.00	0.04	97.64	93.34	43.53
5	Bu_23-6M	4,084,971,264	4,016,865,600	100.00	0.04	97.54	93.13	43.49
6	Bu_23-8M	3,199,770,432	3,125,886,912	100.00	0.04	97.58	93.11	43.60
7	Bu_23-4F	3,241,075,680	3,173,406,912	100.00	0.04	97.76	93.64	43.59
8	Bu_23-7F	5,712,059,808	5,594,345,280	100.00	0.04	97.69	93.46	43.51
9	Bu_23-9F	4,606,916,256	4,527,418,752	100.00	0.04	97.59	93.22	43.75
10	Bu_23-12F	3,494,583,360	3,413,777,472	100.00	0.04	97.54	93.14	43.53
11	Bu_23-15F	3,500,934,624	3,431,798,784	100.00	0.04	97.60	93.25	43.54
12	Bu_23-16F	4,918,103,136	4,844,668,608	100.00	0.04	97.54	93.14	43.50
