# Supplementary material for: Identification of sex-linked molecular markers in the Asiatic toad (Bufo gargarizans Cantor, 1842) reveals an XX/XY sex determination system
Source: BMC Genomics. 2026 Aug 14;27:935. doi: 10.1186/s12864-026-13266-w (PMC13753293; doi:10.1186/s12864-026-13266-w)
Supplement: Supplementary file 3 [file 12864_2026_13266_MOESM4_ESM.pdf]

Table S3. Summary of primers for identification of sex-specific markers in *Bufo gargarizans*

Locus	Primer sequence (5′–3′)	T (℃)	length (bp)	SNP (X/Y)	SNP (Location)
Clocus_23110	Forward: AGCCAACCTACTGACTCTGA<br>Reverse: TGGTTGCTAAGAGTTTCAAAGC	59	195	G/A_A/G	104_144
Clocus_167542	Forward: TGTTGTGGATAGGGAAAGTAGTT<br>Reverse: CAATAATAGTGAAAACGCTCGGTTA	59	260	C/T_G/T_C/A	196_207_238
Clocus_183021	Forward: ACTCTTGGTGCATAGCTTTTGT<br>Reverse: ACCTCTTTAACCCCTTCAGC	59	228	T/G	101
Clocus_345235	Forward: GTCACATCGGGTTACAAAATC<br>Reverse: GTGAAGCAATGACGGAGCA	59	269	A/G_A/C_C/G_C/T	25_37_232_263
Clocus_715040	Forward: ACCTACAGACCAAGCACACT<br>Reverse: TTCCCTCCTTCTTCAGCTGC	59	228	C/T	195
Clocus_1287084	Forward: TGGGATGTCAGTCTTGTGCT<br>Reverse: GTTGTAAGCAGCGGACCAAATAAG	59	264	C/T_G/T	242_243
Clocus_1335887	Forward: GCTATGCTCCCAACCGAAAG<br>Reverse: CCGGGAATCTATCAGGTTCGA	59	208	C/T	202
CLocus_223043	Forward: GCCTCAAGATAACCCCAGTG<br>Reverse: GCCCCACCCAACTCTAGAC	59	250	/	/

Note: The forward and reverse primers were designed at conserved region. T (℃) indicates the annealing temperature of PCR thermal cycling. Length indicates PCR product size. SNP (Location) represents the nucleotide site within the locus, reported using a zero-based offset (first nucleotide is enumerated as 0), and the number represents the locus ID in the catalog.
