# Supplementary material for: Identification of sex-linked molecular markers in the Asiatic toad (Bufo gargarizans Cantor, 1842) reveals an XX/XY sex determination system
Source: BMC Genomics. 2026 Aug 14;27:935. doi: 10.1186/s12864-026-13266-w (PMC13753293; doi:10.1186/s12864-026-13266-w)
Supplement: Supplementary file 4 [file 12864_2026_13266_MOESM5_ESM.pdf]

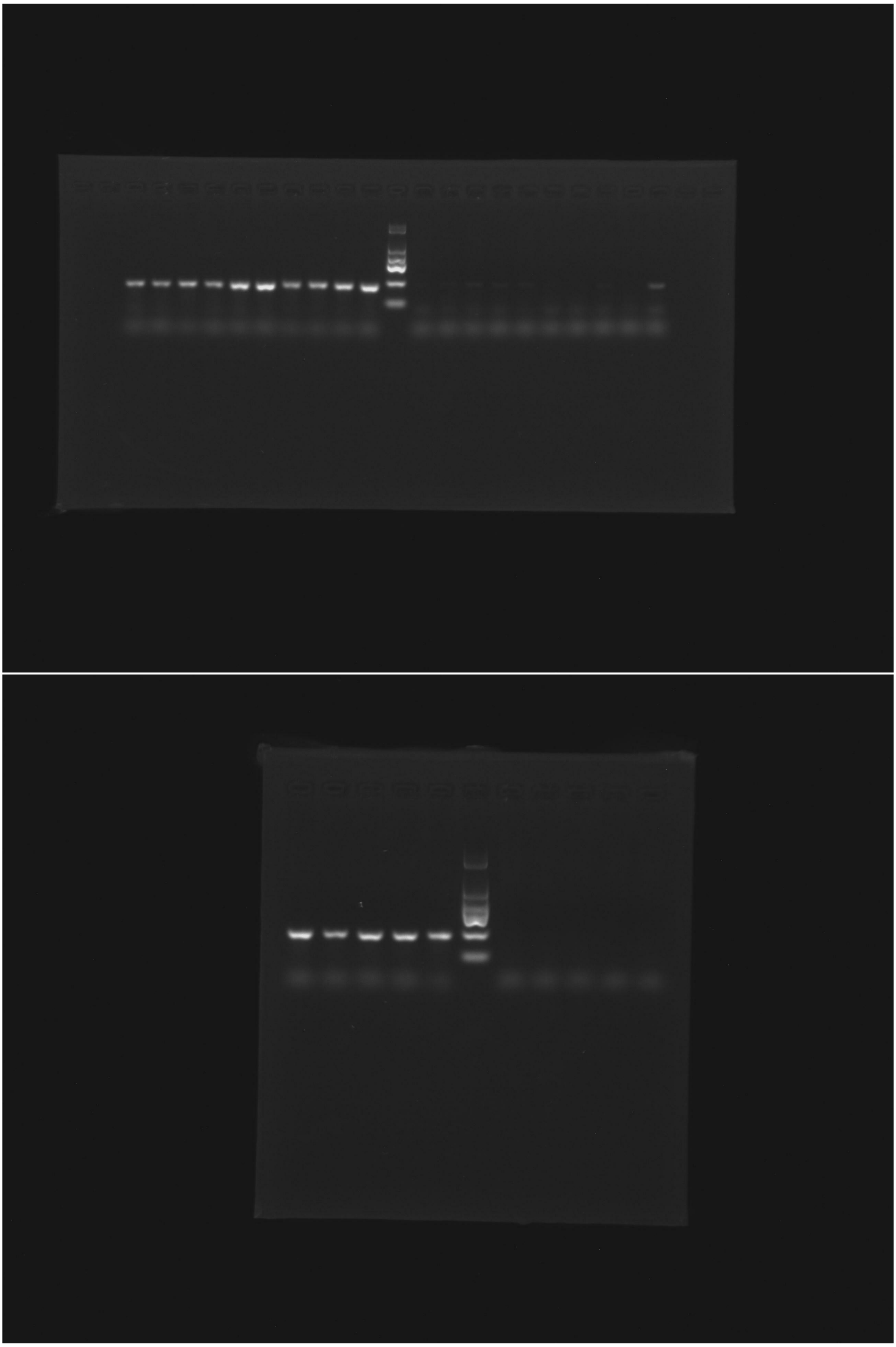

Figure 2B. Full uncropped gel image of PCR validation for Clocus_167542.

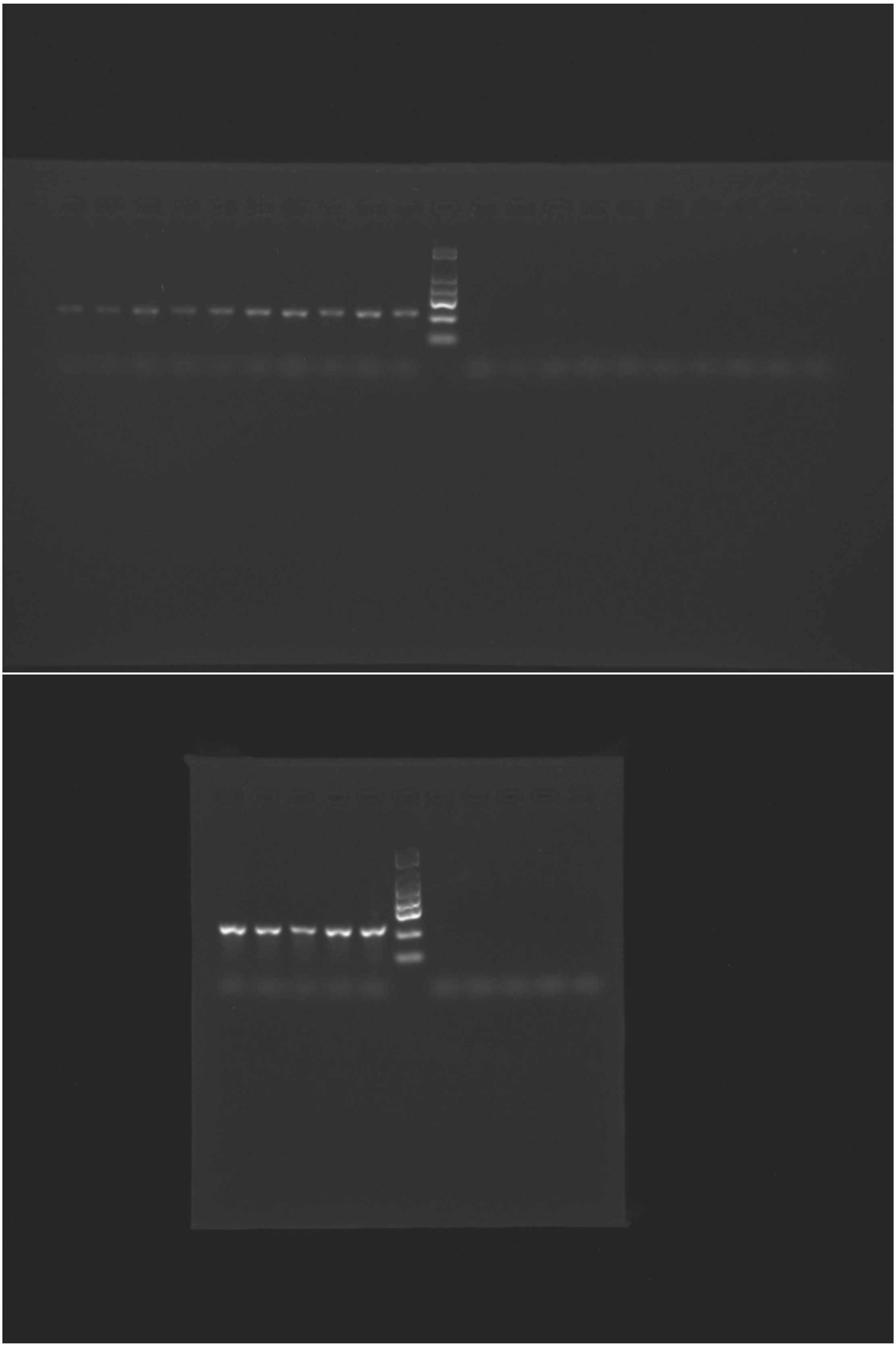

Figure 2C. Full uncropped gel image of PCR validation for Clocus_345235.

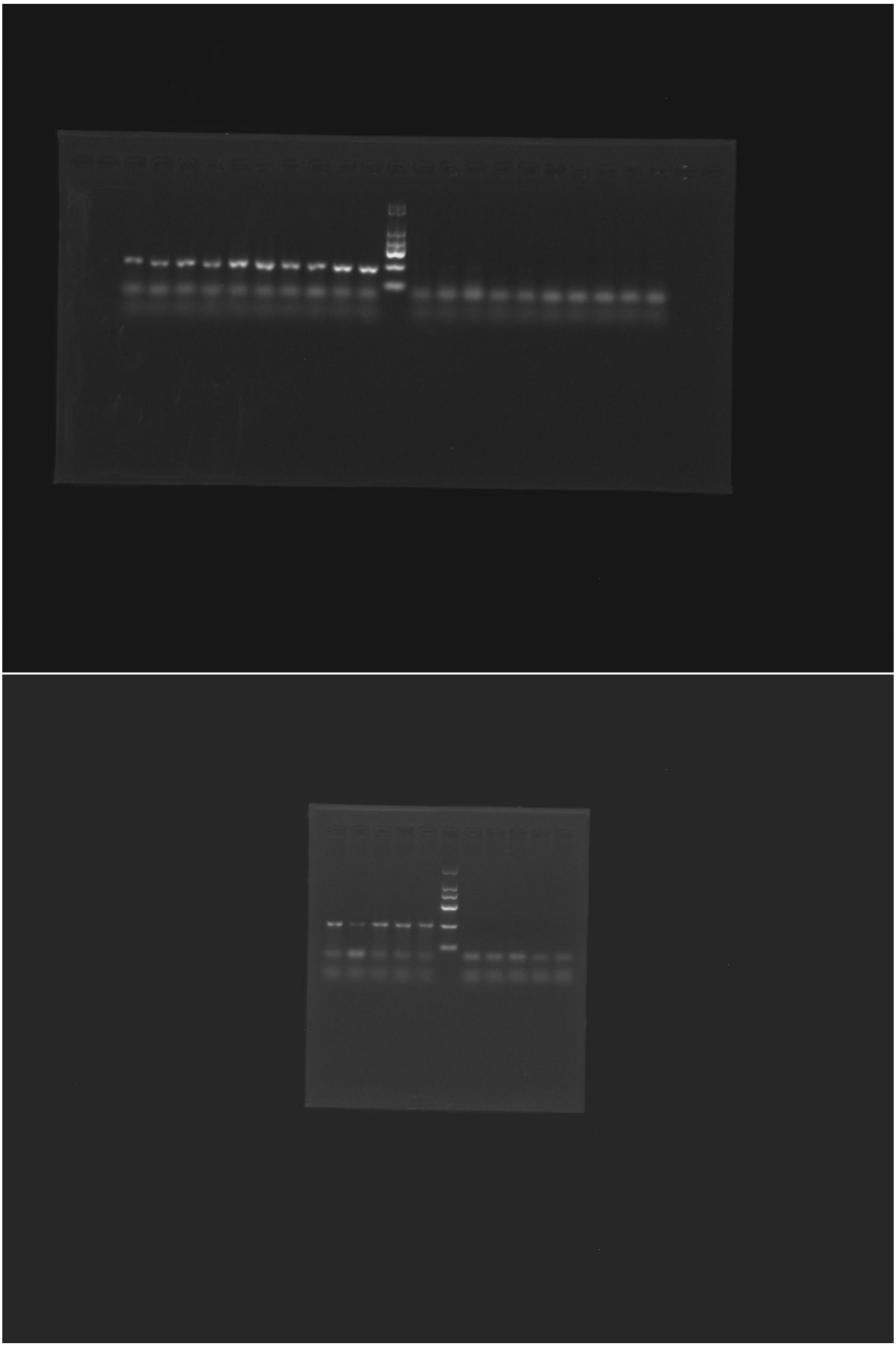

Figure 2D. Full uncropped gel image of PCR validation for Clocus_1287084.

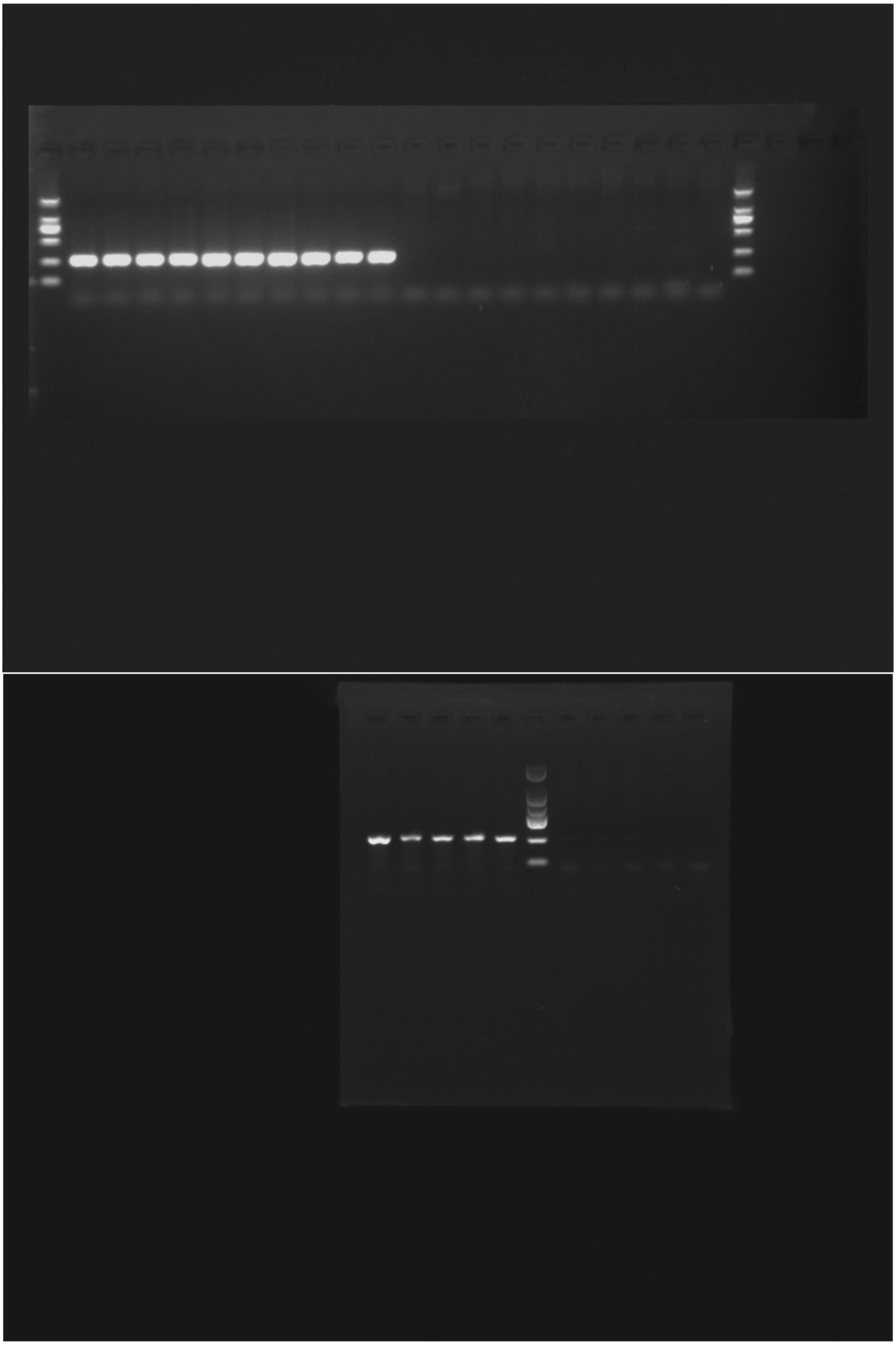

Figure 2F. Full uncropped gel image of PCR validation for Clous223043.
