# Supplementary material for: Identification of sex-linked molecular markers in the Asiatic toad (Bufo gargarizans Cantor, 1842) reveals an XX/XY sex determination system
Source: BMC Genomics. 2026 Aug 14;27:935. doi: 10.1186/s12864-026-13266-w (PMC13753293; doi:10.1186/s12864-026-13266-w)
Supplement: Supplementary file 5 [file 12864_2026_13266_MOESM6_ESM.pdf]

Table S1 Quality summary of *Bufo gargarizans* (6♂，6 ♀) by ddRAD sequencing

Serial	Sample	raw_total_reads	clean_total_reads	Effective Rate(%)	Error Rate(%)	Q20(%)	Q30(%)	GC Content(%)
1	Bu_23-1M	28,884,082	28,235,658	100.00	0.04	97.58	93.23	43.49
2	Bu_23-2M	25,673,238	25,093,192	100.00	0.04	97.61	93.29	43.38
3	Bu_23-3M	21,394,248	20,976,260	100.00	0.04	97.43	92.89	43.56
4	Bu_23-5M	26,782,104	26,013,060	100.00	0.04	97.64	93.34	43.53
5	Bu_23-6M	28,367,856	27,894,900	100.00	0.04	97.54	93.13	43.49
6	Bu_23-8M	22,220,628	21,707,548	100.00	0.04	97.58	93.11	43.60
7	Bu_23-4F	22,507,470	22,037,548	100.00	0.04	97.76	93.64	43.59
8	Bu_23-7F	39,667,082	38,849,620	100.00	0.04	97.69	93.46	43.51
9	Bu_23-9F	31,992,474	31,440,408	100.00	0.04	97.59	93.22	43.75
10	Bu_23-12F	24,267,940	23,706,788	100.00	0.04	97.54	93.14	43.53
11	Bu_23-15F	24,312,046	23,831,936	100.00	0.04	97.60	93.25	43.54
12	Bu_23-16F	34,153,494	33,643,532	100.00	0.04	97.54	93.14	43.50
