# Supplementary material for: Identification of sex-linked molecular markers in the Asiatic toad (Bufo gargarizans Cantor, 1842) reveals an XX/XY sex determination system
Source: BMC Genomics. 2026 Aug 14;27:935. doi: 10.1186/s12864-026-13266-w (PMC13753293; doi:10.1186/s12864-026-13266-w)
Supplement: Supplementary file 6 [file 12864_2026_13266_MOESM7_ESM.pdf]

Supplemental Information

Identification of sex-linked molecular markers in the Asiatic toad (*Bufo gargarizans* Cantor, 1842) reveals an XX/XY sex determination system

Contents

Figure S1. 2
Figure S2. 2
Figure S3. 3
Figure S4. 4
Figure S5. 5
Figure S6. 6
Figure S7. 7
Figure S8. 8
Figure S9. 9
Figure S10. 9
Figure S11. 10
Figure S12. 11
Figure S13. 11
Figure S14. 12
Figure S15. 12

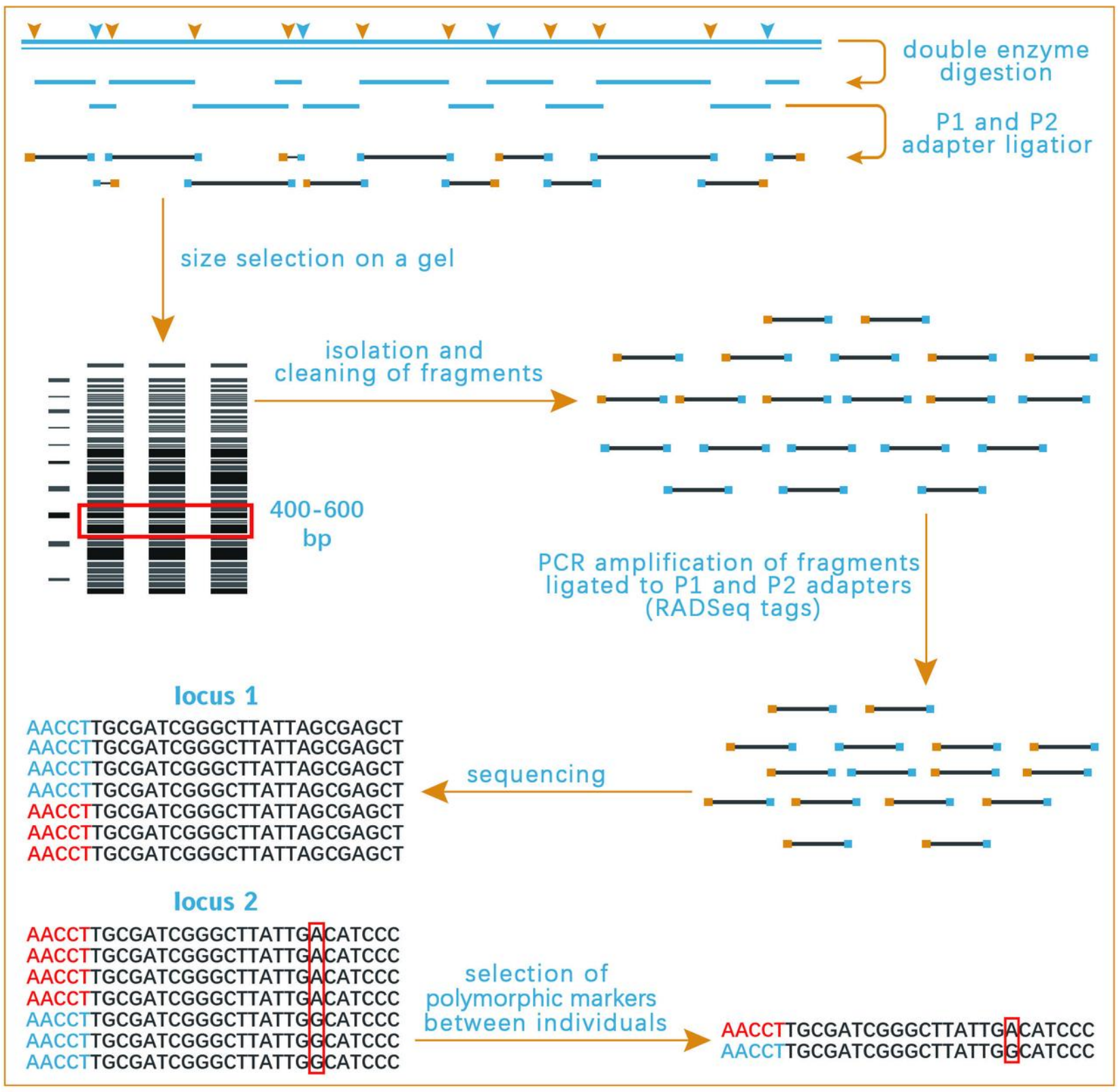

Figure S1. Schematic diagram of the ddRAD experimental workflow.

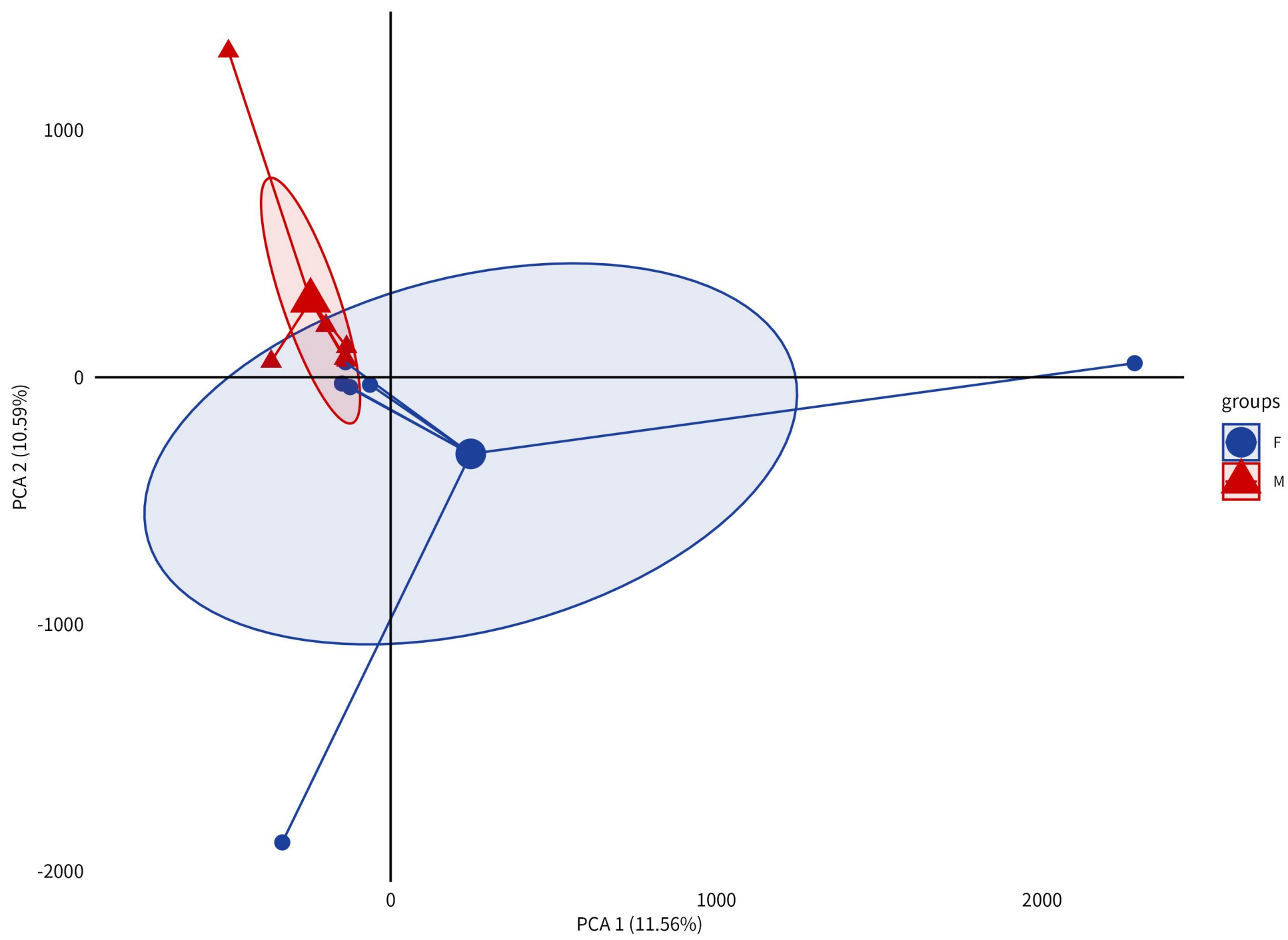

Figure S2. PCA of 12 individuals in two populations based on SNPs present in 100% samples, computed using ADEgenet.

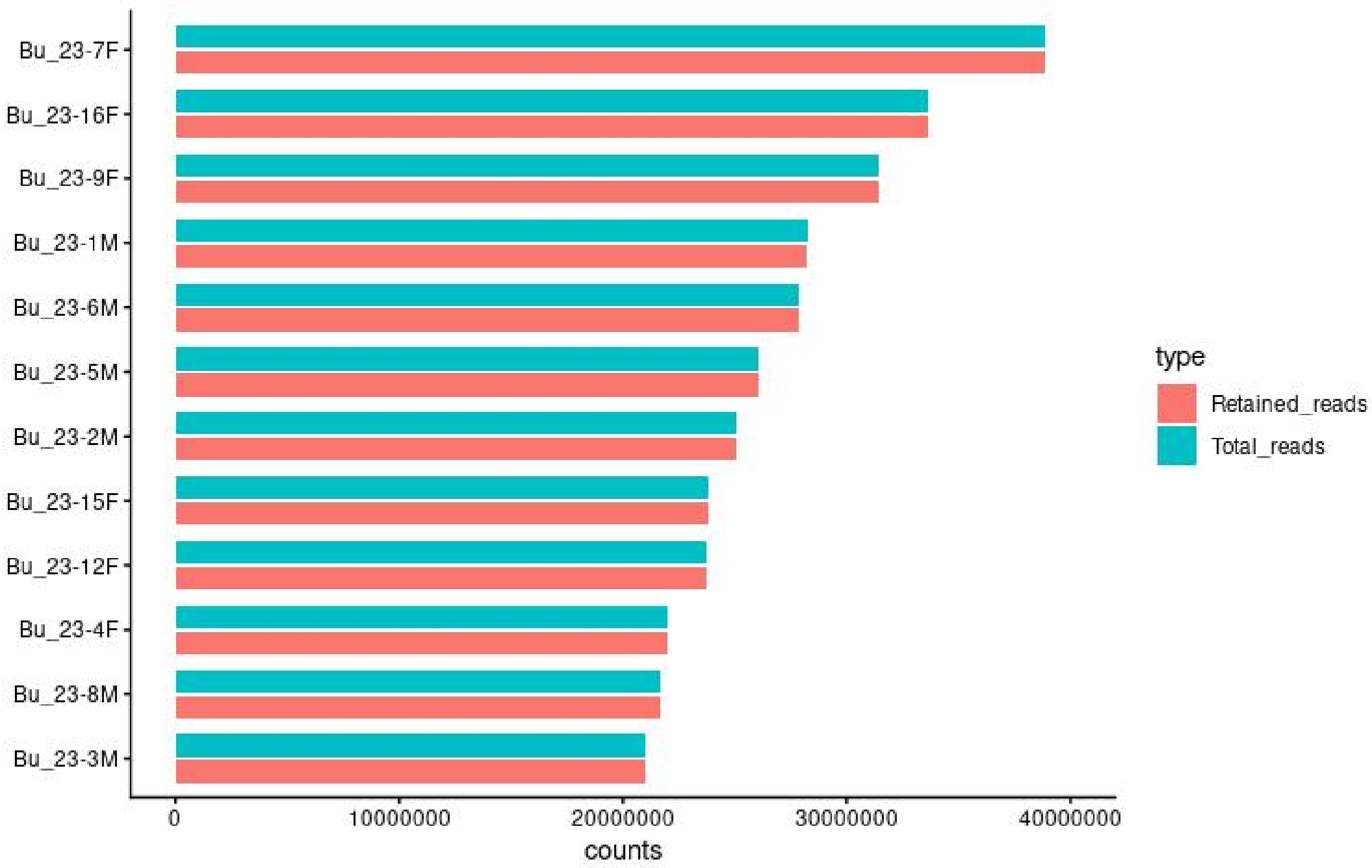

Figure S3. The results from *Process_radtags* of *B. gargarizans*.

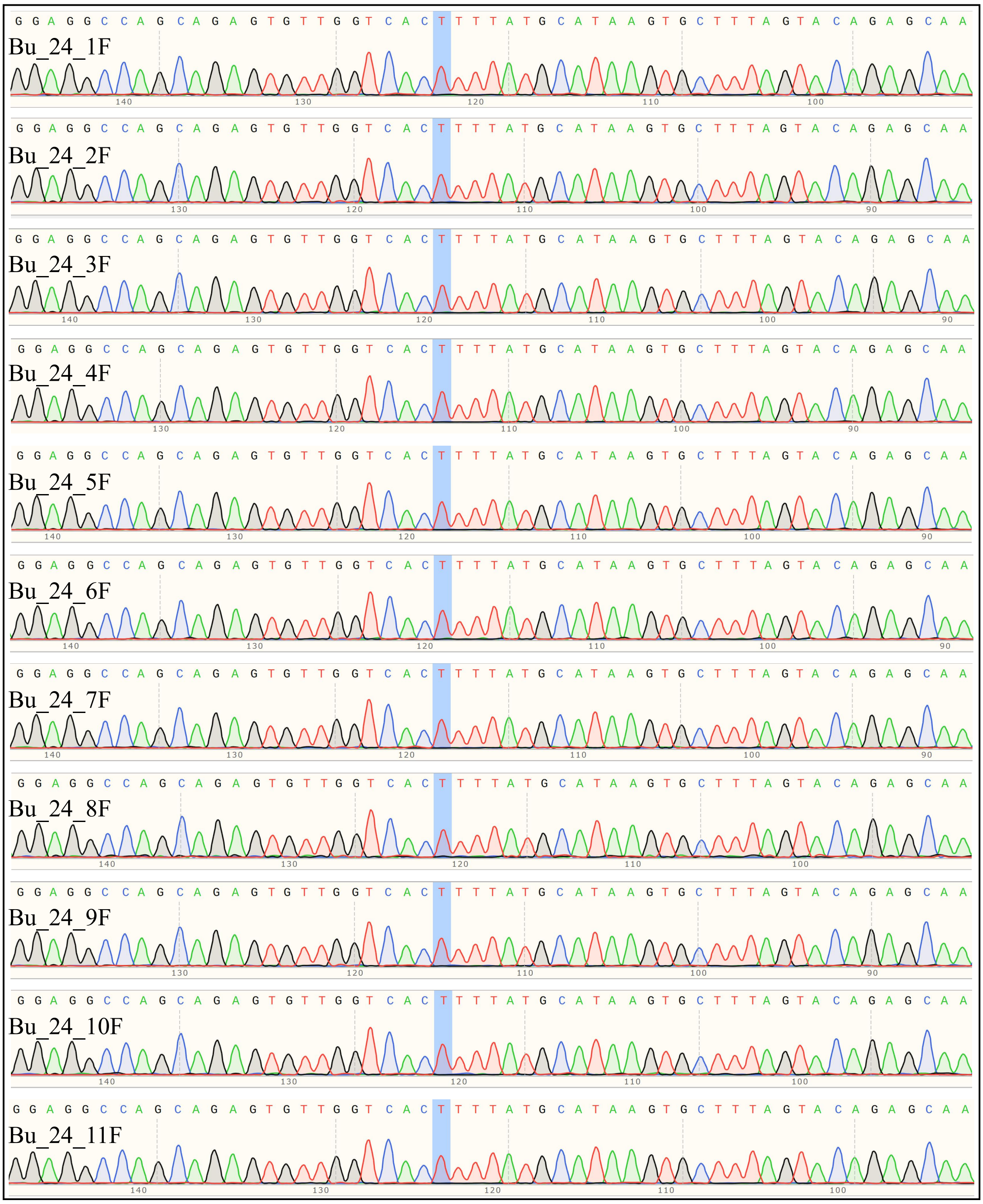

Figure S4 The PCR validation results for the sex-linked marker Clocus_183021. The variable loci were shown in blue boxes. The results indicate that female *B. gargarizans* individuals are homozygous (T/T) at this locus.

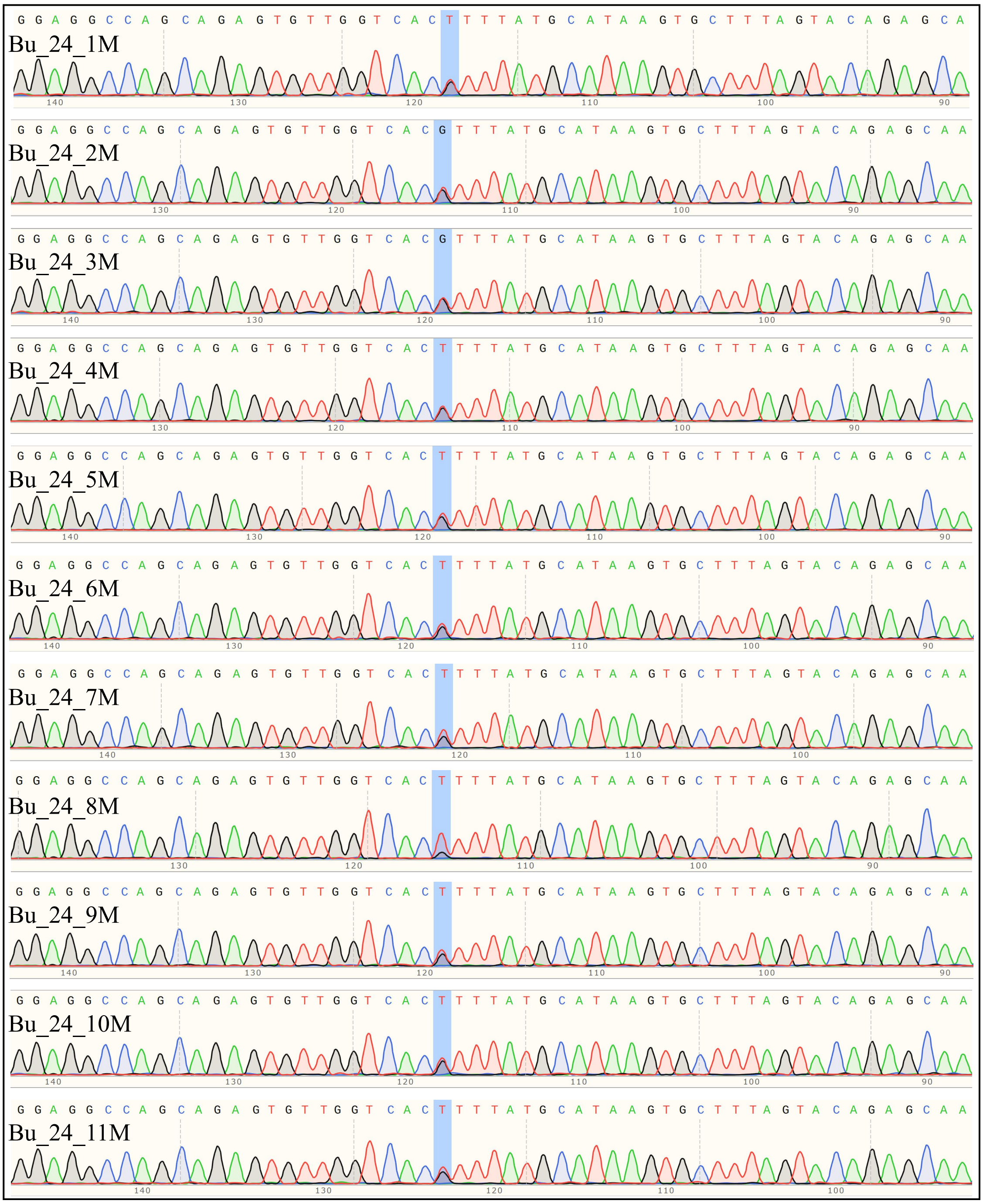

Figure S5. The PCR validation results for the sex-linked marker Clocus_183021. The results indicate that male *B. gargarizans* individuals are heterozygous (T/G) at this locus.

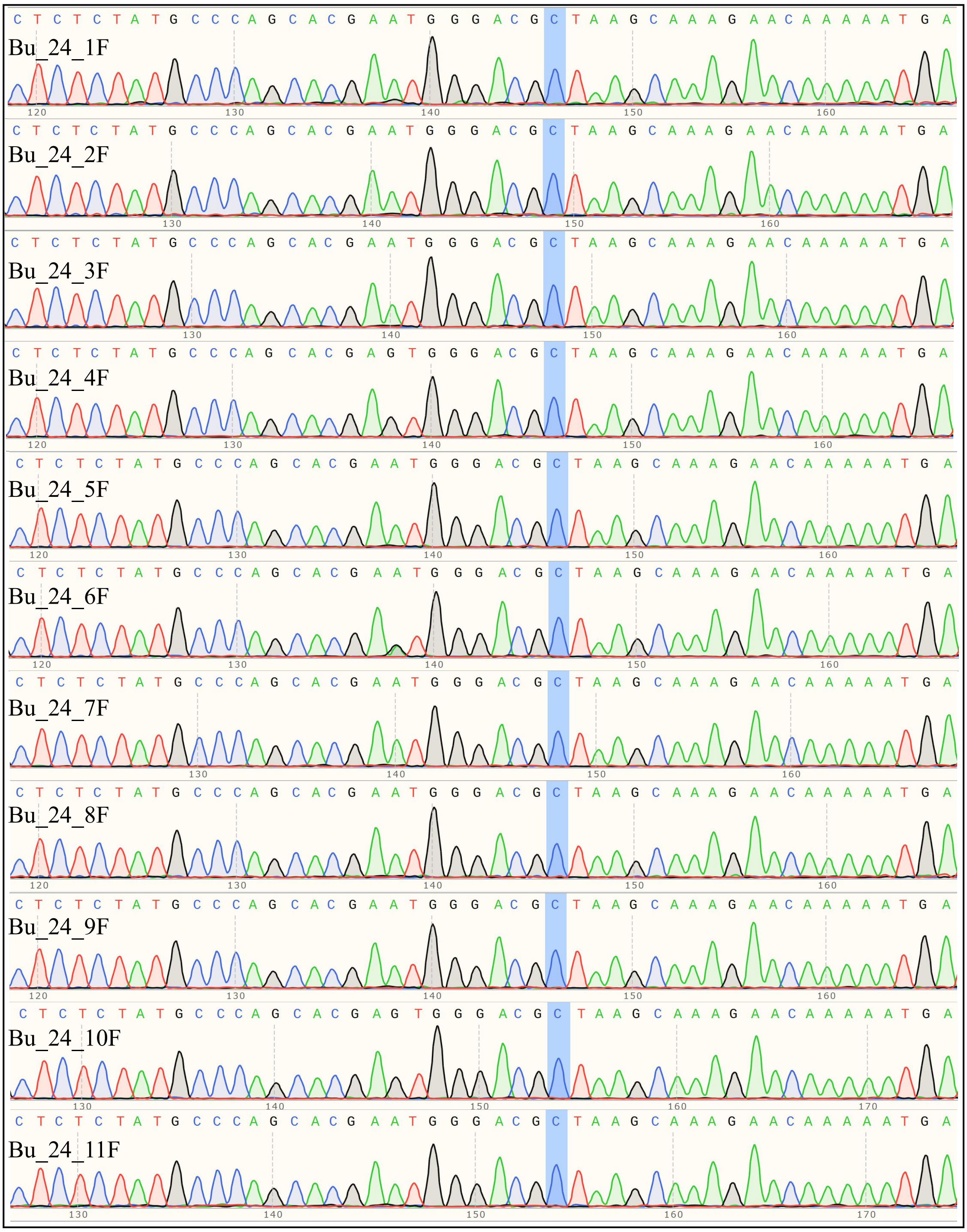

Figure S6. The PCR validation results for the sex-linked marker Clocus_715040. The results indicate that female *B. gargarizans* individuals are homozygous (C/C) at this locus.

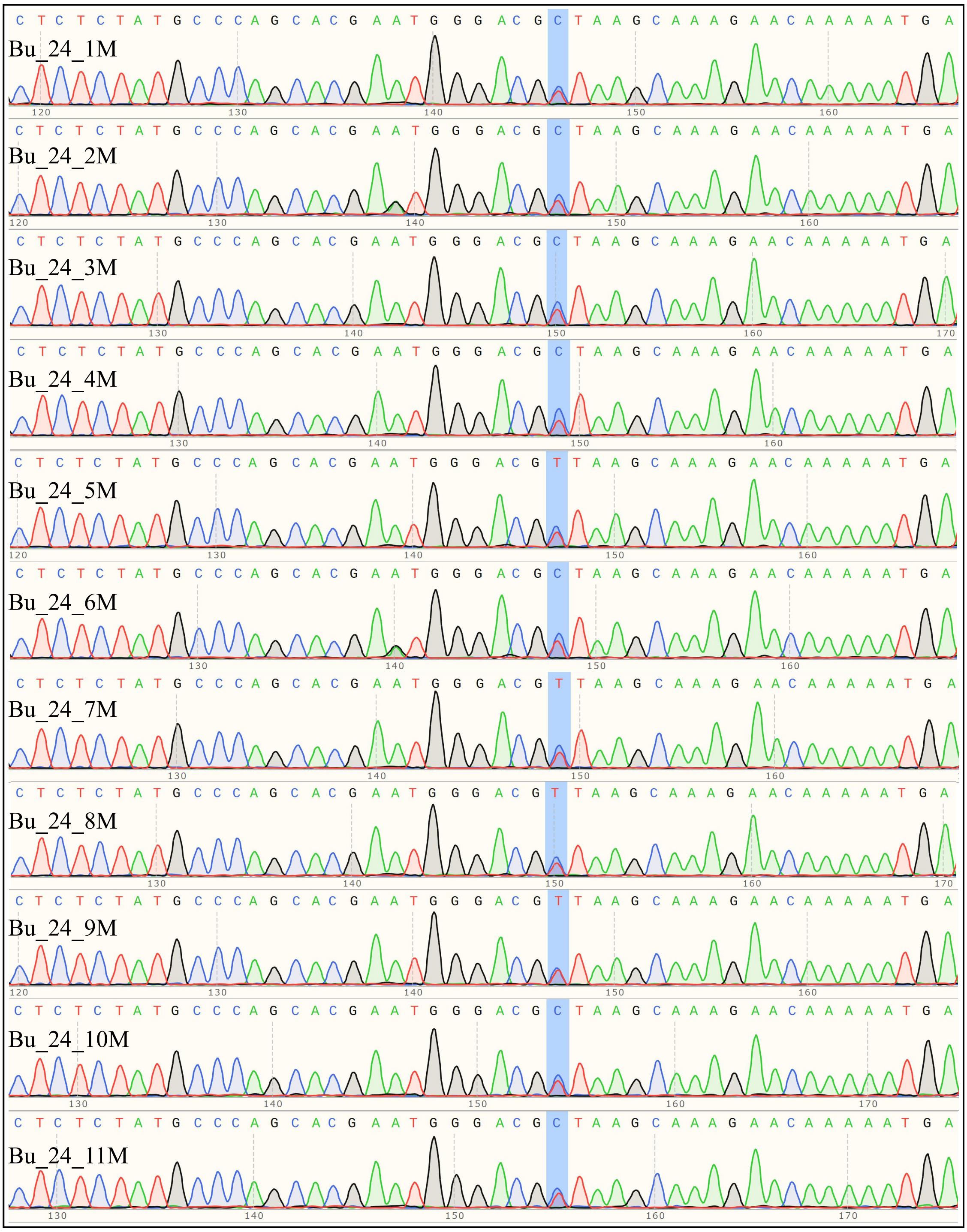

Figure S7. The PCR validation results for the sex-linked marker Clocus_715040. The results indicate that male *B. gargarizans* individuals are heterozygous (C/T) at this locus.

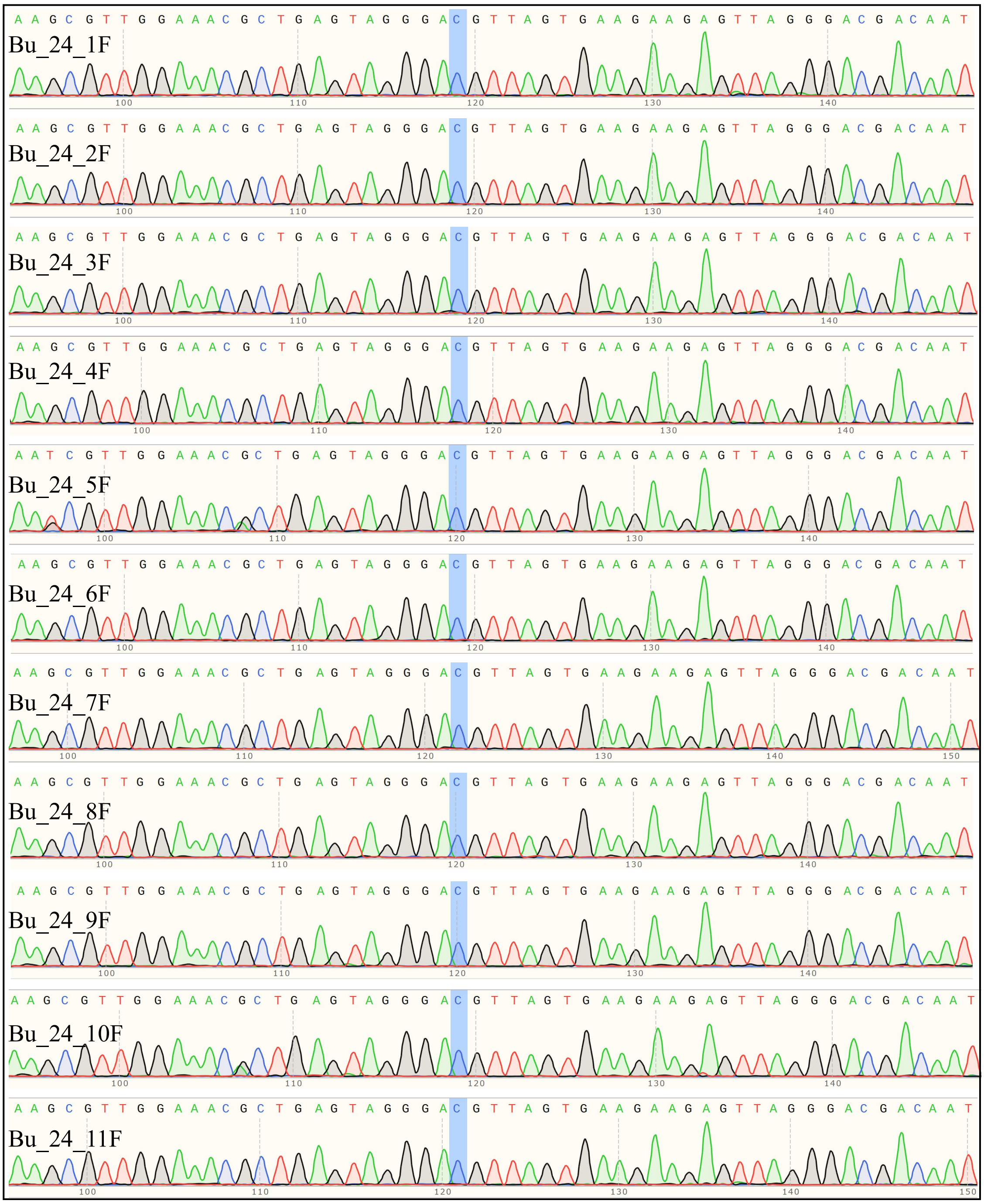

Figure S8. The PCR validation results for the sex-linked marker Clocus_1335887. The results indicate that female *B. gargarizans* individuals are homozygous (C/C) at this locus.

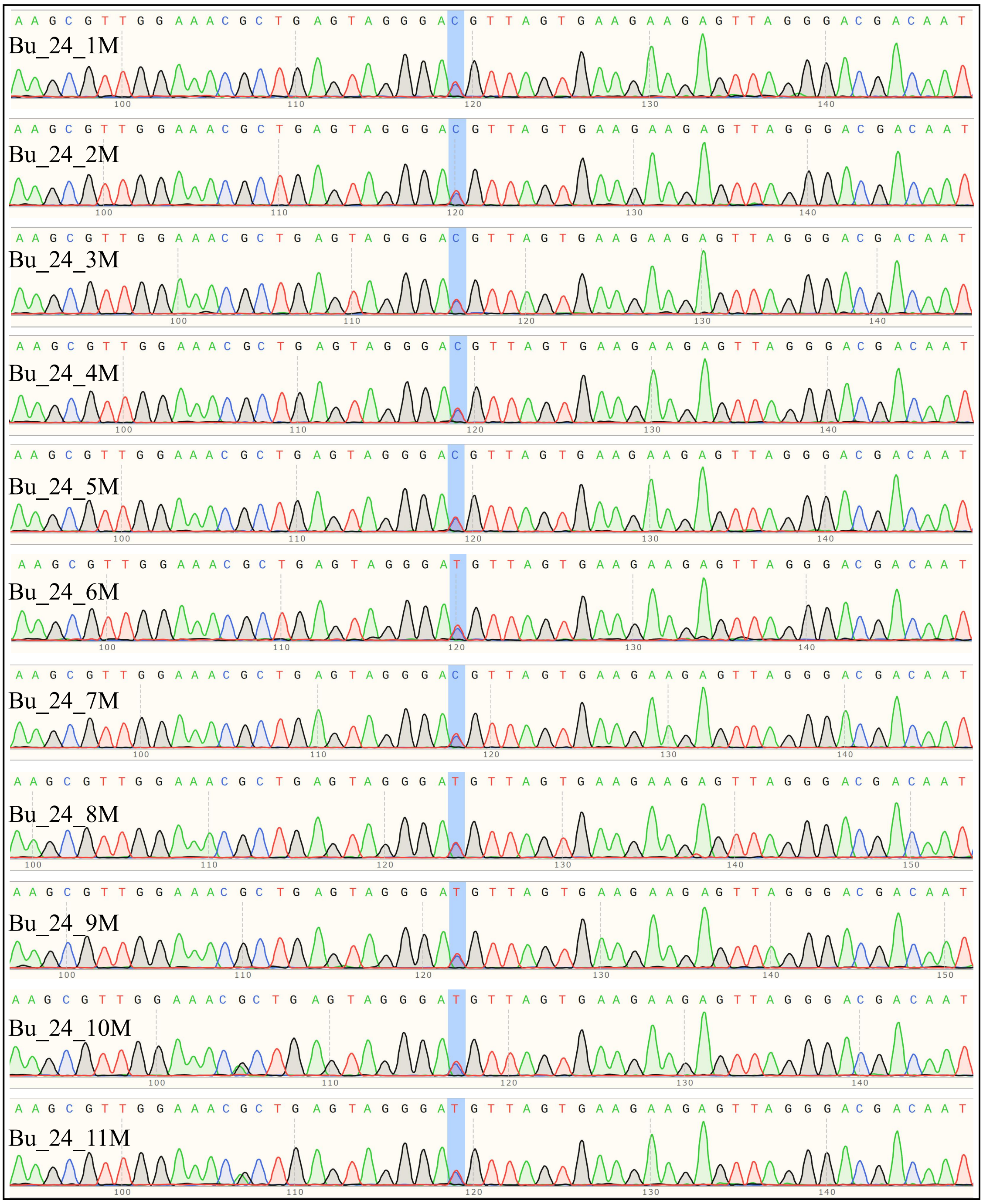

Figure S9. The PCR validation results for the sex-linked marker Clocus_1335887. The results indicate that male *B. gargarizans* individuals are heterozygous (C/T) at this locus.

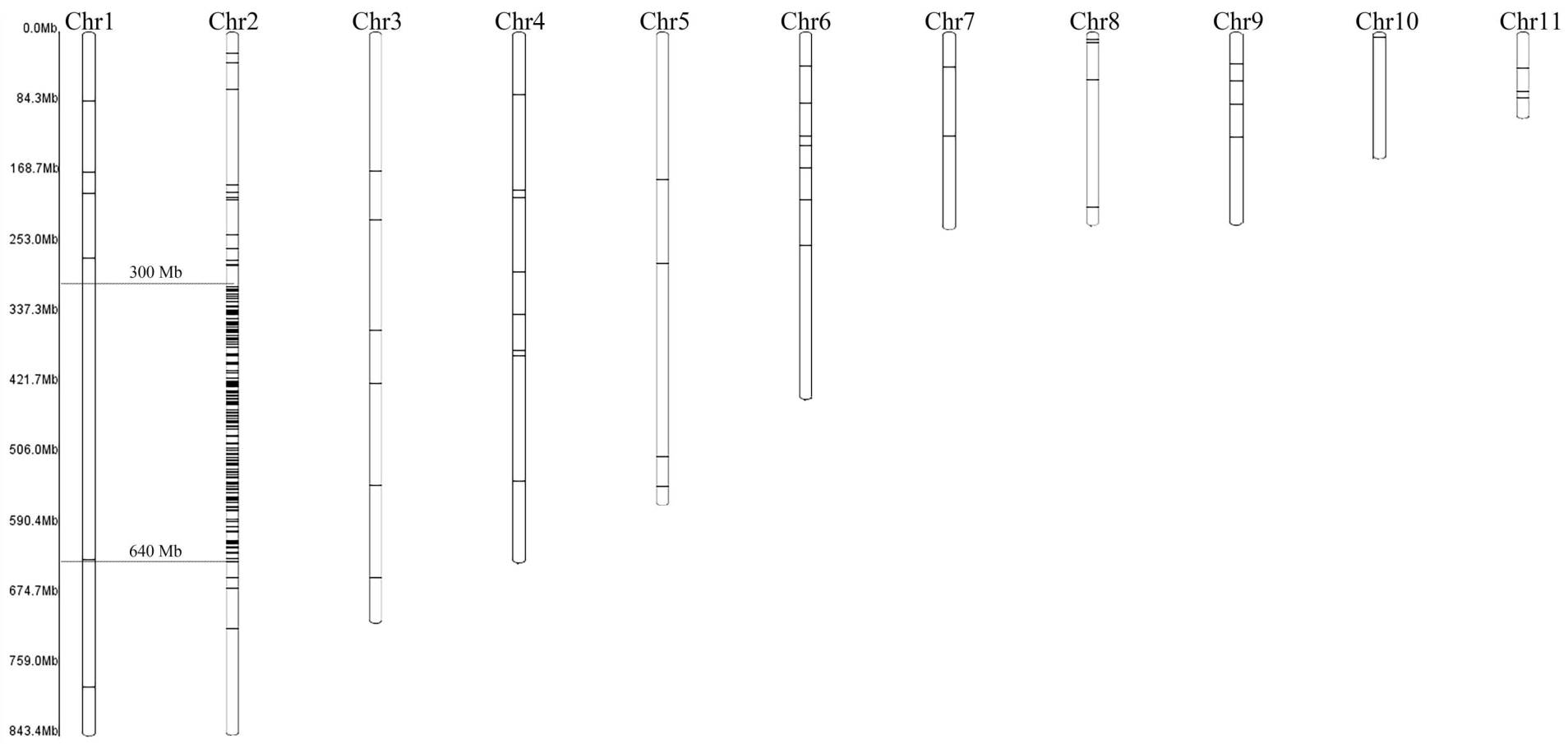

Figure S10. The patterns of sex-linked RAD-tag (SNPs) markers in *B. gargarizans* as compared to the *B. bufo* reference chromosome assembly are described. Sex-linked RAD-tag (SNP) are indicated by small black horizontal lines.

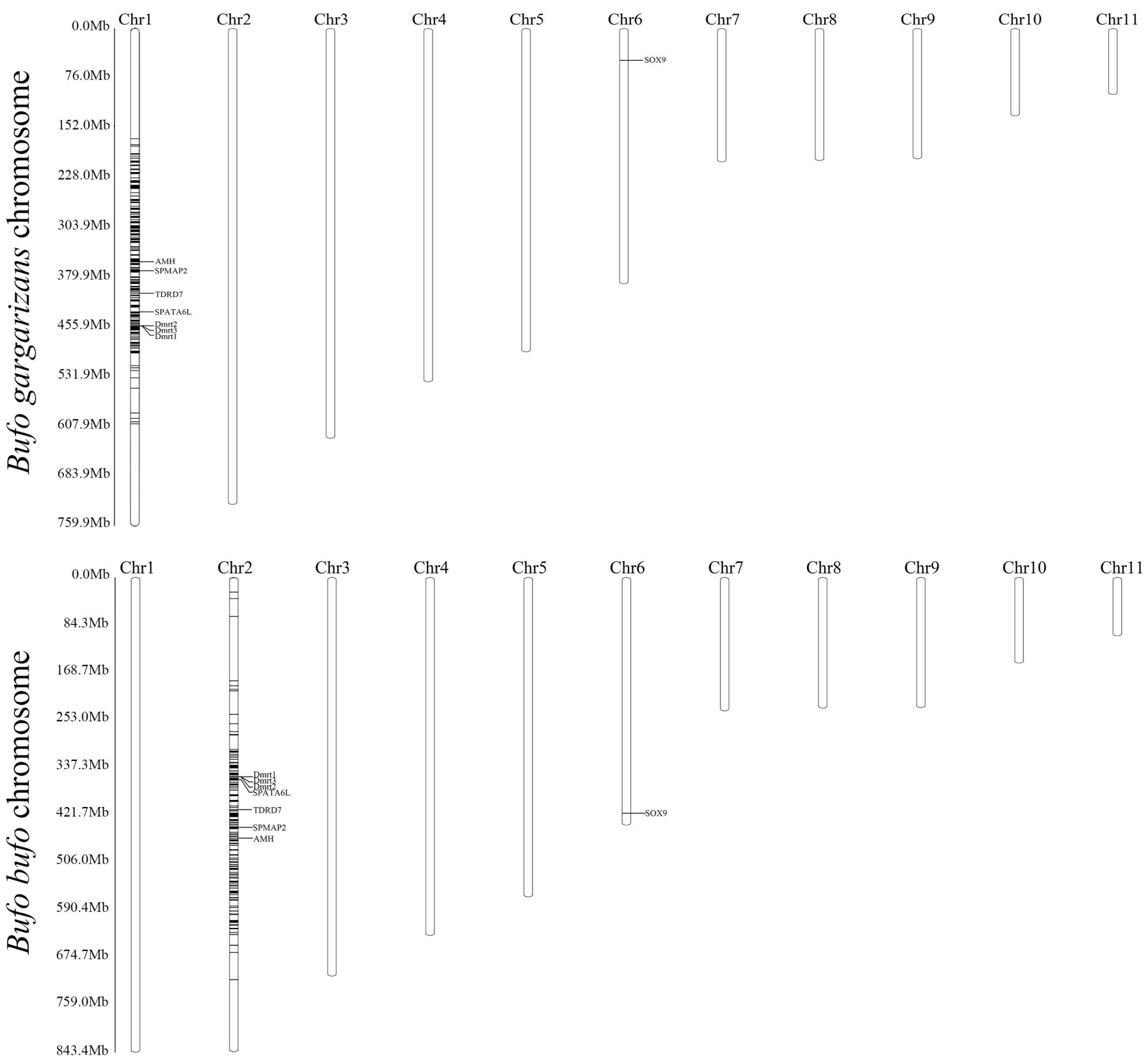

Figure S11. Known genes involved in sex determination, sexual differentiation, or gonad development were mapped onto the reference genomes of *B. gargarizans* and *B. bufo*. Sex-linked RAD-tag (SNP) are indicated by small black horizontal lines.

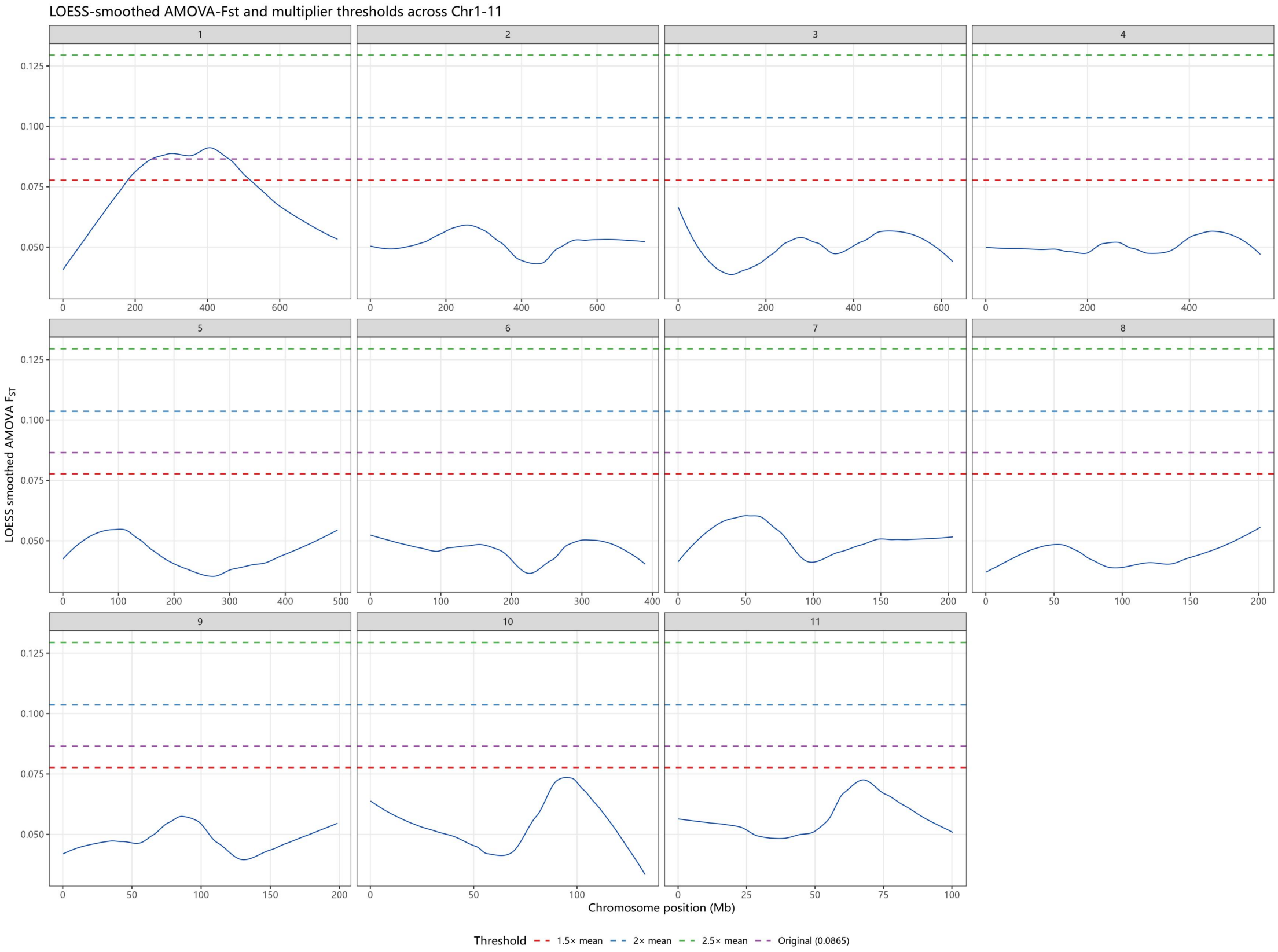

Figure S12. NRR sensitivity multiplier thresholds all chromosome.

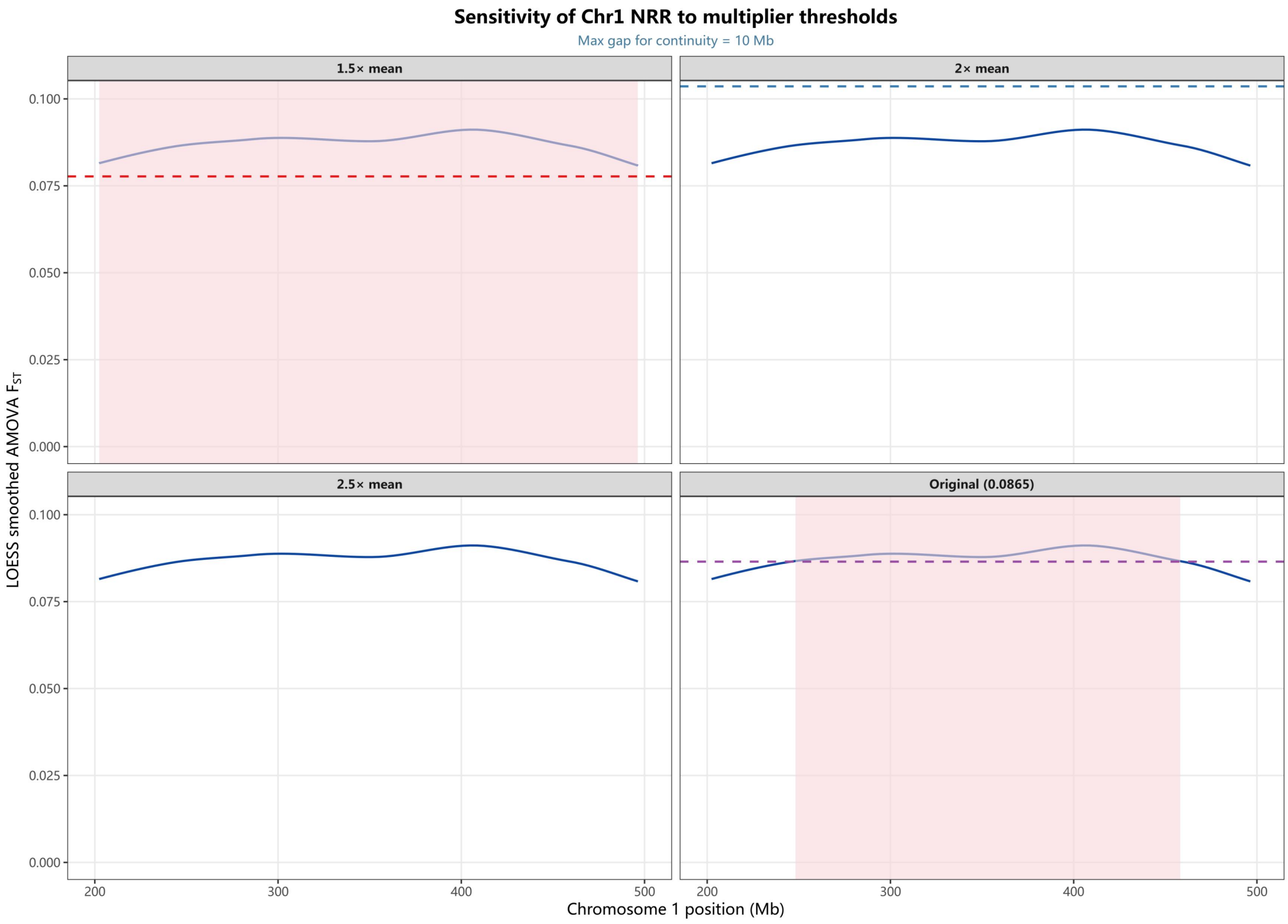

Figure S13. NRR sensitivity multiplier thresholds chr1.

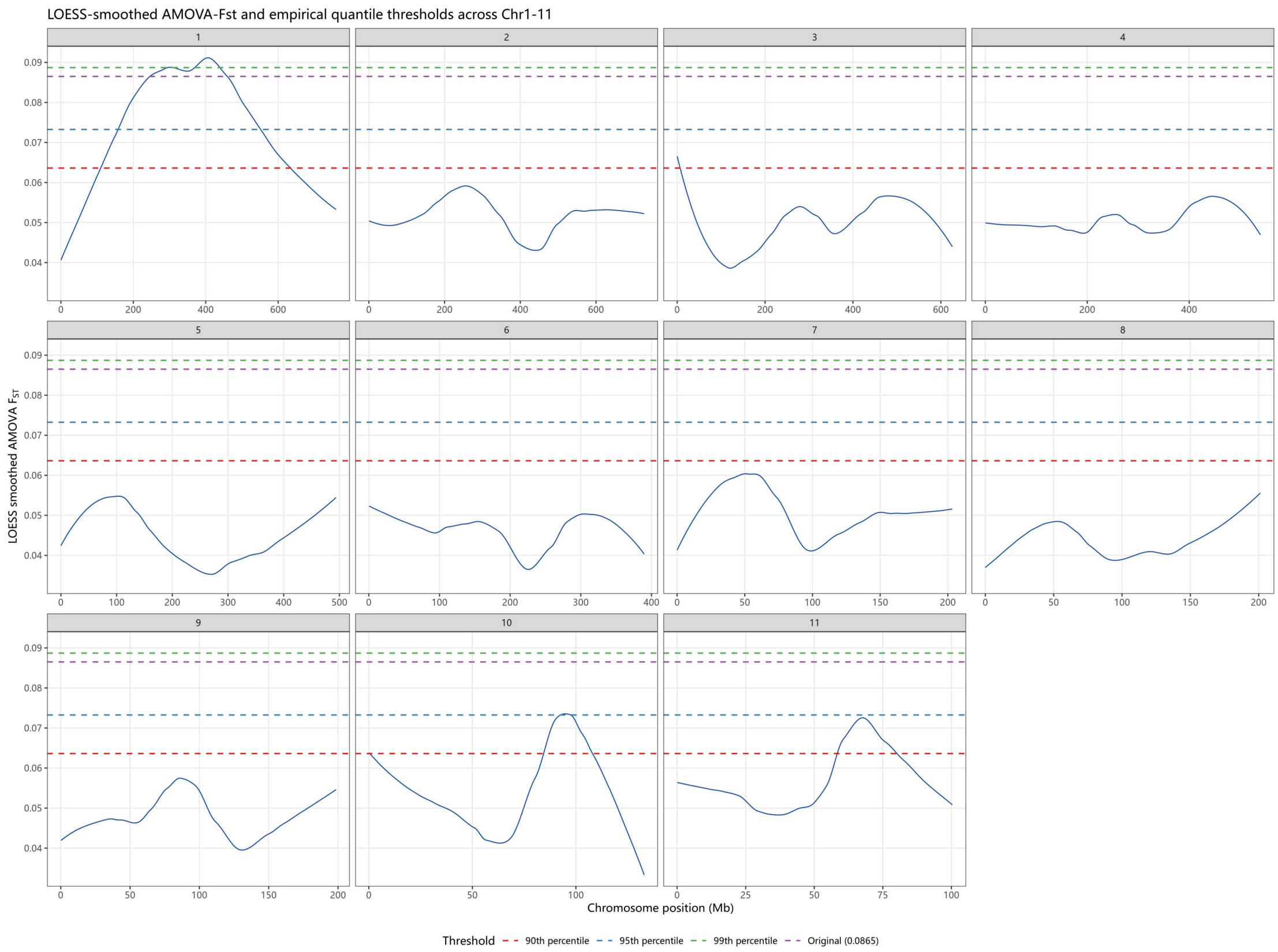

Figure S14. NRR sensitivity quantile thresholds all chromosome.

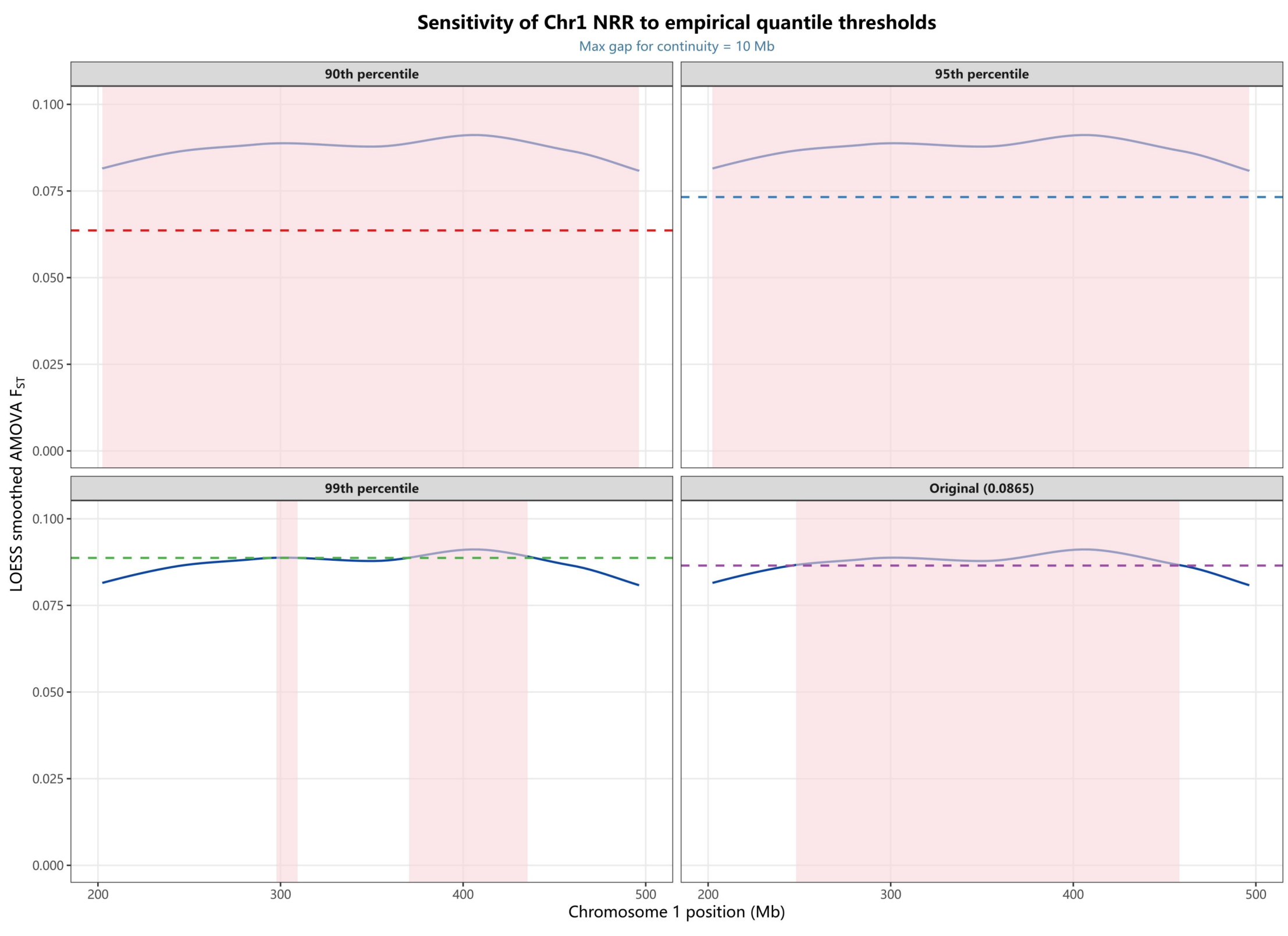

Figure S15. NRR sensitivity quantile thresholds chr1.
